# Study of Geometric Illusory Visual Perception – A New Perspective in the Functional Evaluation of Children With Strabismus

**DOI:** 10.3389/fnhum.2022.769412

**Published:** 2022-04-13

**Authors:** Juliana Tessari Dias Rohr, Cassiano Rodrigues Isaac, Adriano de Almeida de Lima, Ana Garcia, Procópio Miguel dos Santos, Maria Clotilde Henriques Tavares

**Affiliations:** ^1^Department of Ophthalmology, Hospital de Base do Distrito Federal, Brasília, Brazil; ^2^Department of Physiological Sciences, Institute of Biological Sciences, University of Brasília – UnB, Brasília, Brazil; ^3^Department of Ophthalmology, Hospital Regional da Asa Norte – HRAN, Brasília, Brazil; ^4^Faculty of Health Sciences, University of Brasília – UnB, Brasília, Brazil; ^5^Department of Medicine, Unieuro University Center, Brasília, Brazil

**Keywords:** strabismus, children, amblyopia, depth perception (stereopsis), visual illusion, visual perception

## Abstract

Despite the various perceptual-motor deficits documented in strabismus, there is a paucity of studies evaluating visual illusions in patients with strabismus. The aim of this study was to examine how the illusionary perception occurs in children/adolescents (10–15 years old) with strabismus with referral for surgery to correct ocular deviations. A controlled cross-sectional study was carried out in which 45 participants with strabismus and 62 healthy volunteers aged 10–15 years were evaluated. The behavioral response to three geometric illusions [Vertical-Horizontal illusion, Müller-Lyer illusion (Bretano version) and Ponzo illusion] and respective neutral stimuli (non-illusory images) regarding the estimation of image size and response time were measured using the Method of Adjustment. To analyze the influence of secondary factors: type of ocular deviation (convergent, divergent or associated with vertical deviation); amount of eye deviation; presence of amblyopia and stereopsis, a one-way ANOVA was performed. Among the tested illusions, children with strabismus showed greater susceptibility (*p* = 0.006) and response time (*p* = 0.004) to Ponzo’s illusory images. Children with strabismus and preserved stereopsis, on the other hand, showed similar susceptibility and response time to control group patients to the Ponzo illusion (*p* < 0.005). Patients with amblyopia showed overcorrection in the estimate of non-illusory Ponzo images (*p* = 0.046). Children with horizontal ocular deviation (esotropia or exotropia) associated with vertical deviation (hypertropia, DVD and/or alphabetical anisotropy) showed higher susceptibility to vertical adjustment images for the Müller-Lyer illusion (Brentano version) (*p* = 0.017). Individuals with strabismus tended to overcorrect the length of the straight-line segment adjusted for non-illusory images when testing non-illusory images in the Müller-Lyer test (Brentano version) (*p* = 0.009), as well as for the neutral images in the Vertical-Horizontal test (*p* = 0.000). The findings indicated impairment in the perception of geometric illusions and neutral figures, especially for the Ponzo illusion test by children with strabismus. As the behavioral response to illusory images may indirectly reflect the visual and morphofunctional alterations present in these individuals, we suggest that the investigation of visual illusory perception can be used as a new research strategy in the field of investigating the visual function in strabismus.

## Introduction

Strabismus is an eye alignment disorder that affects 2–3% of children worldwide with severe consequences on visual perception as a whole (Gaertner et al., 2013; Kanonidou et al., 2013; Bui Quoc and Milleret, 2014; Hamm et al., 2014; Ridha et al., 2014; Bucci et al., 2016; Ghasia et al., 2018; Milleret and Bui Quoc, 2018). When it affects children at an early age, strabismus can be particularly harmful since the visual system is still in the process of maturating (Milleret and Bui Quoc, 2018).

The lack of synergy in the motor and sensory system to maintain ocular alignment can lead to central morphological structural repercussions such as the volumetric loss of white and gray matter and changes in cerebral blood flow (Xiao et al., 2007; Scholl et al., 2013; Ouyang et al., 2017; Tan et al., 2018). Functional alterations such as cortical suppression and amblyopia are also reported (Webber and Wood, 2005; Hamm et al., 2014). Eye movement disorders such as the increase in ocular fixation instability and the increase in the disconjugacy of fixational saccades and intrasaccadic ocular drift are also seen in patients with strabismus, even in the absence of amblyopia and latent nystagmus (Bucci et al., 2002; Ghasia et al., 2018). Amblyopia patients with strabismus and individuals with deficits in stereoscopic vision also show slow response to sensorimotor range tasks (reach performance), as well as motor inaccuracy in locating targets in space with positional uncertainty and perceptual image distortions (Niechwiej-Szwedo et al., 2017). More recently, a slow reading ability (Ridha et al., 2014) and difficulties in postural control (Gaertner et al., 2013; Bucci et al., 2016) have been documented, in addition to the considerable psychological and socio-emotional impact that has already been widely discussed (Schuster et al., 2019).

To ensure proper image perception, the eyes must be align so that the world projected onto the retinas falls on both foveae (Coubard et al., 2014; Otero-Millan et al., 2014; Parr and Friston, 2017; Walton et al., 2017; Ghasia et al., 2018). The information acquired is initially encoded by the retina in both eyes and ascends through the visual pathways until it reaches the Central Nervous System, where it will be organized and interpreted based on the individual’s previous experiences in order to give meaning to the presented stimulus (Coubard et al., 2014; Parr and Friston, 2017). This means that, from the visual stimulus, the individual will build up a scene from their point of view, which ultimately determines their interaction with the environment that surrounds them (Otero-Millan et al., 2014).

As such, it is understood that visual perception is an essential core activity of human life (Parr and Friston, 2017). An important study tool in the behavioral field to assess visual perception is illusory images (Eagleman, 2001). Visual illusions have been used in Neuroscience as a first step in research to investigate neural image processing (Gori et al., 2016). Illusory figures are commonly defined as images in which reality is perceived incorrectly, where there are discrepancies between what is experienced by the individual and what is physically expressed (Eagleman, 2001). However, for Shapiro and Hedjar (2019), this definition does not reflect the complexity that involves the visual experience. In fact, the perception we have of a stimulus is not a true copy of reality. The authors reinforce the concept that the brain is the “reality engine” capable of integrating all aspects of visual perception to build a consistent “reality.” As such, illusions could be re-defined as conditions in which there would be a conflict between the possible interpretations of a perceptual “reality” (Shapiro and Hedjar, 2019). That way, visual illusions represent a powerful window to access the functional integrity of the central nervous system, as they prove that our view of the world comes from continuous interpretations at a central level (Eagleman, 2001; Coubard et al., 2014; Axelrod et al., 2017; Parr and Friston, 2017; Shapiro and Hedjar, 2019).

There is a wide variety of illusory images. In the literature, illusions of shape, color, contrast, movement, after-image, impossible figures, among others, are described (Hamburger et al., 2007; Gori and Yazdanbakhsh, 2008; Ninio, 2014; Hamburger, 2016). In addition to their applicability in basic research, visual illusions are widely used in studies in population groups with medical conditions with central repercussions such as: autism, dyslexia (Chouinard et al., 2016; Gori et al., 2016; Manning et al., 2017), and schizophrenia (Grzeczkowski et al., 2018; Costa et al., 2021). Investigations in the field of illusory images have provided important clues about neural architecture that contribute to the understanding of the principles of central visual processing (Eagleman, 2001). The study of the Hermann Grid Illusion, for example, contributed to the theory of lateral interaction between neuronal cells, helping to understand neural connections and circuits of excitation and inhibition at the central level (see Eagleman, 2001, for more details).

Behavioral (Cretenoud et al., 2019, 2020), electrophysiological (Senkowski et al., 2005) and morphofunctional studies (He et al., 2015; Axelrod et al., 2017) have been carried out to understand the cerebral areas and neural mechanisms involved in illusory visual perception. However, it is still debatable at what level visual illusions are processed and what would be the determining factors that underly mechanisms of visual illusions (Cretenoud et al., 2020). Factors such as the individual’s age (Cretenoud et al., 2020), cross-cultural and educational aspects (Leibowitz et al., 1969), visual context in which the illusion is inserted (Cretenoud et al., 2020), as well as aspects such as: shape, color, texture, luminance and orientation of images (Cretenoud et al., 2019) have been studied.

According to Cretenoud et al. (2020), there are multiple factors that influence illusory visual perception and vision in general. Their results demonstrate, for example, a slight general decrease in susceptibility to Ponzo and Müller-Lyer illusions with age, a fact that does not correlate with the context in which the images are inserted. The authors also reported a weak correlation in the magnitude of susceptibility between the illusions tested. However, there was strong intra-illusion correlation (when considering presentation variations under different contexts of the same illusion). These results indicate that there is no common factor for visual illusions (Cretenoud et al., 2020).

Currently, an attempt is made to understand the visual functionality and perceptual changes experienced by patients with strabismus (Ridha et al., 2014; Pineles et al., 2015; Bucci et al., 2016). However, there are only a few studies that assess behavioral aspects and visual illusions in this population group. Considering the morphological anomalies at the central level perceptual changes in patients with strabismus, some questions arose that prompted this study to be carried out: (1) Are individuals with strabismus susceptible to geometric visual illusions? (2) Are there differences in the illusory visual perception of individuals with strabismus when compared to individuals without strabismus? In view of the functional alterations already confirmed by other studies, we could assume that, yes, strabismus would present alterations in illusory visual perception.

For our research, we chose three classic geometric illusory images with robust illusion magnitude (the Vertical-Horizontal, the Brentano version of the Müller-Lyer illusion, and the Ponzo illusion). Geometric visual illusions form a heterogeneous group of two-dimensional figures that require complex central processing (Ninio, 2014; Cretenoud et al., 2019) and high-level perceptual interactions (top-down regulation) with a predominant influence in contextual interpretive processing (Yildiz et al., 2021).

The aim of our study was to evaluate the susceptibility (in millimeters) and response time (in seconds) of patients with strabismus, with a referral for surgery to correct ocular deviation, for these three visual illusions and their respective neutral images (non-illusory) and compare the results with the response of healthy individuals. Our results demonstrate that patients with strabismus have greater susceptibility and response time, especially to the geometric Ponzo visual illusion, compared to individuals in the Control Group. Furthermore, we demonstrate that the lack of stereopsis (three-dimensional vision) impacts Ponzo’s illusory perception.

The study of visual illusions in individuals with strabismus constitutes a new and relevant approach to the assessment of the functionality of the visual system. Our work opens the door to countless possibilities for future studies using, for example, the association of behavioral and morpho-functional techniques (such as Nuclear Magnetic Resonance and Electrophysiology). In addition, we propose that visual illusions can be used as a basis to compare and monitor possible central remodeling arising from the improvement of ocular alignment with the treatment of surgical correction of strabismus (Gori et al., 2016).

## Materials and Methods

### Participants

The study sample consisted of 107 children/adolescents, divided into two groups: The Cases Group (individuals with strabismus with a referral for surgery to correct ocular deviation) and the Control Group (healthy individuals without strabismus). The individuals with strabismus were categorized into three subgroups according to the classification of the ocular deviation presented: individuals with convergent deviation (Esotropias), individuals with divergent deviation (Exotropia) and individuals with an association of horizontal deviation with vertical gaze deviation (Dissociated Vertical Deviation - DVD or Hypertropia) and/or alphabetic anisotropies (Hertle, 2002).

For the Cases Group, 62 individuals were examined, and 45 children/adolescents were selected, aged 10–15 years (mean 11.96 ± 1.65) with strabismus and with ocular deviation less than or equal to 60 prism diopters, with referral for surgery to correct strabismus in the Ophthalmology sector of Hospital Regional da Asa Norte, Brasília. Seventeen individuals with surgical strabismus who did not meet the established inclusion criteria were excluded from the sample, as follows: five patients with residual strabismus (already operated on prior to the study), one patient with ptosis, one patient with keratoconus, two patients with associated congenital cataract, one patient with retinal detachment, one patient with microcephaly, three individuals with mental retardation, two patients with Attention Deficit Hyperactivity Disorder, one patient with Down’s Syndrome. Of the 45 patients included, thirty-two patients (71%) had Esotropia, and thirteen individuals (29%) had Exotropia. Twenty-six individuals (58%) had an association of horizontal deviation with vertical gaze deviation. Visual acuity was tested using a Snellen chart positioned 5 m away, monocularly, with a better visual correction including the use of glasses, if necessary. The size of the ocular deviation was measured, with the best visual correction using the Prism Cover or Krimsky test, and classified as small (if up to 15 diopters), medium (from 15 to 30 diopters) and large (if greater than 30 diopters) (Ghasia et al., 2018). Stereopsis was tested for using the Titmus test, where true stereopsis was considered if better than or equal to 100 s of arc (Lal and Holmes, 2002). The clinical and ophthalmological characteristics of the sample are shown in detail in Supplementary Table 1.

Healthy volunteers were selected after an eye examination had been carried out on 116 students from the public education system in the Federal District. Individuals with Central Nervous System Diseases were prevented from participating in the study. Also, individuals with clinically conductive strabismus and/or with other associated ocular comorbidities, such as cataracts, retinal dystrophies, glaucoma, among others, could not participate. Along with individuals who used drugs with a narcotic effect. The control group included 62 healthy individuals aged 10–15 years (38 girls, 61%), with a mean age of 10.89 years ± 1.05 and ophthalmological examination without changes namely: visual acuity equal to or greater than 8/10 in both eyes without the need for optical correction (using the Snellen chart at 5 m), absence of ocular deviation (orthotropic), with stereopsis of 40 s of arc (Titmus test).

The study was approved by the Research Ethics Committee of the University of Brasília (UnB) (CAAE: 83515717.7.0000.0030) and is part of the first phase of the Clinical Trial registered at the Rebec (Brazilian Registry of Clinical Trials) under number RBR-4tzjjc3 for the evaluation of geometric illusory visual perception in strabismus patients before and after eye alignment surgery. All participants and their legal representatives signed the Informed Consent Form before starting the tests.

### Apparatus and Procedure

**FIGURE 1:**
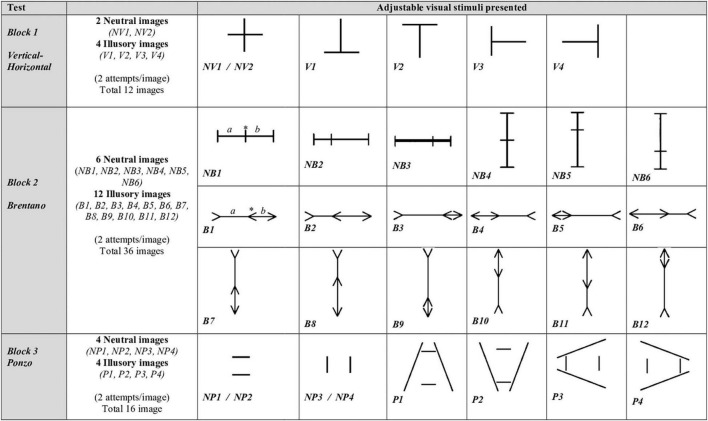
Adjustable visual stimuli presented. Number, type of image (illusory and neutral) and name of the figures shown in each block. Three blocks of images were presented (the Vertical-Horizontal test, the Brentano version of the Müller Lyer test and the Ponzo test). Illusory and neutral images were presented randomly within the three test blocks with an interval of 1 min between the blocks and two repetitions per image, totaling 64 figures per experimental session. To perform the Method of Adjustment, the individual would have to compare the length of the straight segments. If deemed necessary, the individual could adjust one of the straight segments so that they have the same subjective length. For the Vertical-Horizontal Test the vertical straight line should be compared to the horizontal one. The individual could increase or decrease the length of the horizontal (*NV1, V3*, and *V4*) or vertical (*NV2, V1*, and *V2*) straight line of the image. For the Brentano test, the individual could modify the position of the central arrow (in illusory images) or the central straight line (in neutral images), in order to make both line segments (straight segments “a” and “b”) subjectively equal on the main axis of the figure. The central element (*) could be moved freely in the direction of the horizontal axis (to the right or left of the image, in the figures*: NB1, NB2, NB3, B1, B2, B3, B4, B5*, and *B6*) or in the direction of the axis vertical (up or down in figures: *NB4, NB5, NB6, B7, B8, B9, B10, B11*, and *B12*). *NB1*, lines at 90°. Central line on the point of objective equality. *NB2*, lines at 90°. Central line displaced to the left by 18.66 mm. *NB3*, lines at 90°. Central line displaced to the right by 18.66 mm. *NB4*, lines at 180°. Central line on the point of objective equality. *NB5*, lines at 180°. Central line displaced up by 18.66 mm. *NB6*, lines at 180°. Central line displaced down by 18.66 mm. *B1*, arrow pointing right. Central arrow on the point of objective equality. *B2*, arrow pointing right. Central arrow displaced to the left by 18.66 mm. *B3*, arrow pointing right. Central arrow displaced to the right by 18.66 mm. *B4*, arrow pointing left. Central arrow on the point of objective equality. *B5*, arrow pointing left. Central arrow displaced to the left by 18.66 mm. *B6*, arrow pointing left. Central arrow displaced to the right by 18.66 mm. *B7*, arrow pointing down. Central arrow on the point of objective equality. *B8*, arrow pointing down. Central arrow displaced up by 18.66 mm. *B9*, arrow pointing down. Central arrow displaced down by 18.66 mm. *B10*, arrow pointing up. Central arrow on the point of objective equality. *B11*, arrow pointing up. Central arrow displaced down by 18.66 mm. *B12*, arrow pointing up. Central arrow displaced up by 18.66 mm. For the Ponzo test, the individual can modify the length of one of the straight segments in the direction of the horizontal (*NP1, NP2, P1*, and *P2*) or vertical (*NP3, NP4, P3*, and *P4*) axis of the image. *NP1*, top horizontal line adjustable. The individual should compare the length of the top horizontal line with the bottom one and adjust the top horizontal line (increasing or decreasing) so that both horizontal lines have the same subjective length. *NP2*, bottom horizontal line adjustable. *NP3*, right vertical line adjustable. *NP4*, left vertical line adjustable. *P1*, top horizontal line adjustable. *P2*, bottom horizontal line adjustable. *P3*, left vertical line adjustable. *P4*, right vertical line adjustable.

A Positivo laptop was used (Intel^®^ Celeron^®^, Dual-Core) 15′′ 1,366 × 768 LCD monitor for data collection. Visual stimuli were developed in the Eye Lines software™ (Beagley, 1993). Three geometric illusions (the Vertical-Horizontal, the Brentano version of the Müller Lyer and the Ponzo illusion) and their respective neutral (non-illusory) images were presented to the volunteers in black, in the center of the monitor screen on a white background. Specifications regarding the formatting of figures are shown in the Supplementary Table 2. As an adjustment (in the horizontal or vertical direction) and the line to be adjusted for each image presented are shown in Figure 1.

Experimental sessions were conducted binocularly with participants sitting comfortably facing the monitor, keeping a fixed distance of 52 cm from the screen and wearing their glasses, if necessary. When asked, they reported a clear vision and that they were comfortable to perform the experiment. To assess the behavioral response, the Method of Adjustment was used, as described below.

Three blocks of images were presented (the Vertical-Horizontal test, the Brentano version of the Müller Lyer test and the Ponzo test) as shown in Figure 1. Illusory and neutral images were presented randomly within the three test blocks with an interval of 1 min between the blocks and two repetitions per image, totaling 64 figures per experimental session (see Figure 1). For each figure presented, only one of the straight-line segments could be adjusted. The adjustable straight-line segment was predetermined by the examiner when the images where prepared in the Eye Lines software™ (Beagley, 1993). For each figure presented, the straight-line segment located in the vertical or horizontal direction could be adjusted, as shown in Figure 1. To evaluate the figures (neutral and illusory), the individual needed to carefully and quickly estimate the length of the straight segments subjectively and adjust the size of the images using the arrows on the computer keyboard, and if need be, make them subjectively equal in terms of length.

For the neutral images in the Vertical-Horizontal block, individuals could adjust the length of the straight line located vertically or horizontally. For the neutral images in the Brentano block, the individual could adjust the position of the central line of the image in order to make both line segments subjectively equal on the main axis of the figure. For the neutral images of the Ponzo block, the individual could adjust the length of the straight line located vertically or horizontally. For the illusory images in the Vertical-Horizontal block, the individual could adjust the length of the vertical line or the horizontal line of the image. For the illusory images in the Brentano block, the individual could adjust the portion of the central arrow of the figure in order to equalize the length of the two straight segments of the main axis of the image. For Ponzo’s illusion figures, the individual could adjust the size of the horizontal or vertical line segment near the angle that closes the image. To view the adjustable straight-line segment of each image (neutral and illusory) tested see Figure 1. The illusory effect was determined by the actual length of the line segments and the estimation of the line length according to the individual’s subjective perception. After each figure had been evaluated, the individual pressed the “Enter” key to move on to the next image to be evaluated. There was no limitation on image adjustment size or response time for each figure.

The procedure was divided into two Phases, the Training Phase and the Testing Phase. In the Training Phase, individuals were presented with the stimuli, familiarized with the Method of Adjustment and with the keyboard commands (arrow keys and “Enter” key) to change the images. During the Training Phase, all tested images were presented to the volunteers (see Figure 1). We also explained how and which straight line image (illusory and neutral) should be adjusted if the individual deemed it necessary. Data acquired in the Training Phase were not used for statistical analysis purposes. During the Test Phase, interaction between the examiner and the patient was avoided to only access the visual processing of the images evaluated. All sessions were recorded by audio and video to identify possible signs of fatigue whilst carrying out the test. This was not found at any time during the tests.

### Variables Studied

Susceptibility and response time to the visual stimuli presented were evaluated using the Method of Adjustment. The susceptibility or magnitude of the illusory perception was measured using the difference in the size estimated by all participants versus the objective size of the stimulus presented in millimeters (mm). For the response time variable (in seconds), the period of time that elapsed from the moment the image appeared on the screen to the moment when the individual pressed the “Enter” button to move to a new image was considered.

### Statistical Analysis

The mean of the values of the responses regarding susceptibility and time to evaluate and adjust the image was calculated. Mean values were analyzed using the Student’s t test, considering the type of stimulus presented (illusory or neutral) for the Cases and Control Groups. To verify the homogeneity of variance between the groups, we performed the Levene test. When variances were not similar, we used the Welch t test. To separately analyze the influence of each of the secondary factors: type of ocular deviation (convergent, divergent or associated with vertical deviation); amount of eye deviation; the presence of amblyopia and stereopsis, a one-way ANOVA was used. We initially measured which comparisons to the null hypothesis (which states that there is no difference between exposure variables on the outcome in the population) was more likely than the alternative hypothesis. To minimize the possibility of a type I error (False Positive), we used the Bonferroni correction for Tukey’s *Post Hoc* or Honestly Significant Difference (HSD) test. The Shapiro-Wilks test showed deviations from normality equally present in both Groups (Cases and Controls). The significance level for the tests was established as *p* < 0.05. If each type of test (Vertical-Horizontal, Brentano and Ponzo) is considered separately, there are 9 (nine) dependent variables per test. Considering a multiple comparison per test there is a 36.9% chance of obtaining at least one “significant” *p*-value (<0.05) just by chance. The generalized result for independent data is generally weaker than if observed as part of an analysis that involves multiple comparisons. That could be a limitation in our study and the comparisons should be taken with care. However, to fulfill the objectives of the study, an individual analysis was used to discover which illusional perception tests could be more appropriate for individuals with strabismus. Interpreting a set of comparisons is not straightforward. If multiple comparisons have not been corrected, erroneous and “significant” results may be found. On the other hand, if multiple comparisons have been corrected, then real differences may not be detected. In this first phase of our study, we only performed simple comparisons analyzing the effect of each exposure separately on the outcome. All comparisons are shown below in the “Results” section.

## Results

**FIGURE 2:**
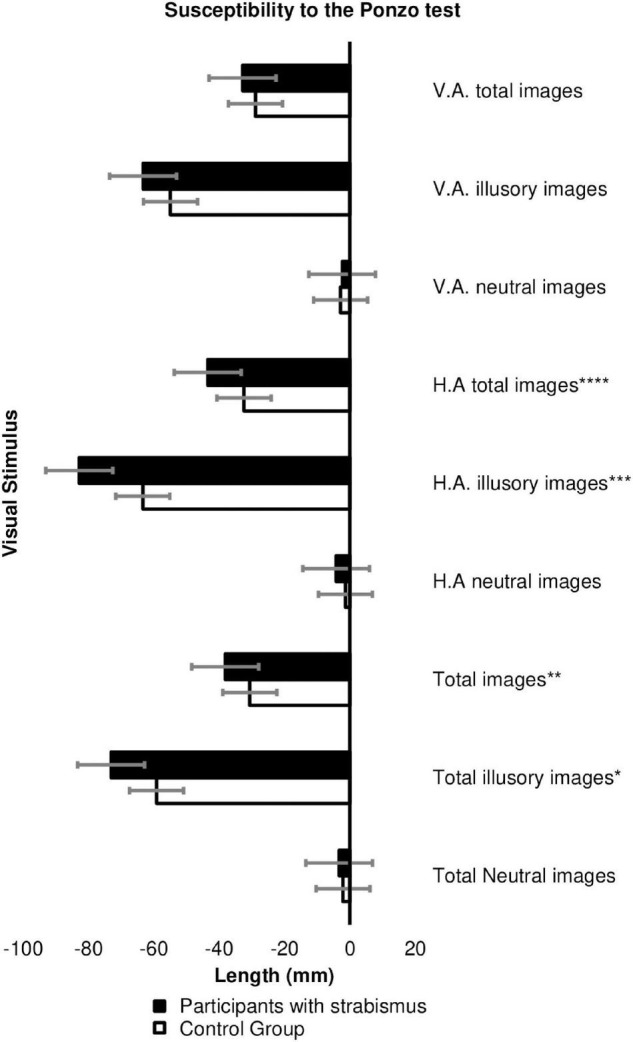
Estimate of the image size (in millimeters) between the Cases (*n* = 45) versus Controls Groups (*n* = 62) in the Ponzo test. HA, horizontal adjustment images; VA, vertical adjustment images. *p-value = 0.030; **p-value = 0.0037, ***p-value = 0.006; ****p-value = 0.005. The error bars shown represent the Standard Error.

In the Ponzo test, individuals with strabismus were more susceptible to estimate the size of the illusory images with horizontal adjustment (*p* = 0.006) compared to the control group. The estimate of volunteers with strabismus was −82.82 ± 36.75 versus −63.38 ± 34.14 mm in the Control Group. Patients with strabismus are also more susceptible for Ponzo illusory images in general (*p* = 0.030) specifically for the horizontal axis adjustment figures (*p* = 0.005; Figure 2 and Supplementary Table 3).

**FIGURE 3:**
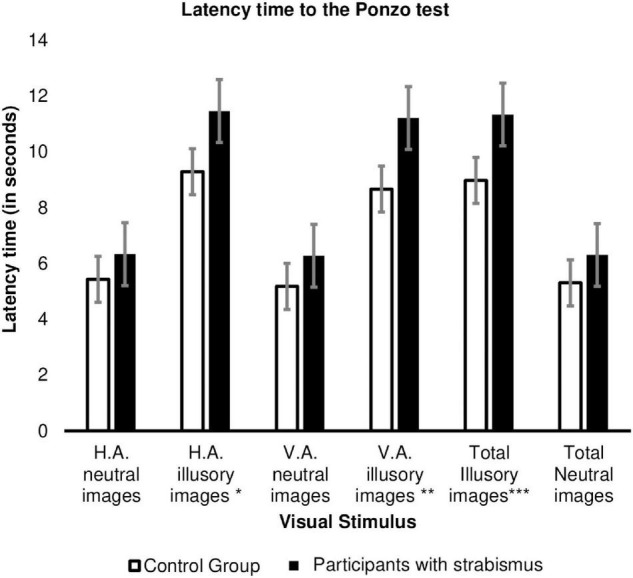
Response time (in seconds) to adjust the image between the Cases (*n* = 45) versus Control Group (*n* = 62) in the Ponzo test. HA, horizontal adjustment images; VA, vertical adjustment images; *p-value = 0.007, **p-value = 0.008, ***p-value = 0.004. The error bars shown represent the Standard Error.

Figure 3 shows a significant difference, with a slow response in adjustment by patients with strabismus to Ponzo illusory images with a horizontal adjustment of 11.460 ± 4.697 s for patients with strabismus versus 9.287 ± 3.521 s for the Control group (*p* = 0.007). Likewise, note the slow response time in the patients with strabismus in the images of vertically adjusted images (11.212 ± 5.990 s for the Cases group versus 8.661 ± 3.793 s for the Controls group; *p* = 0.008) and in illusory images in general (*p* = 0.004; Supplementary Table 3).

**FIGURE 4:**
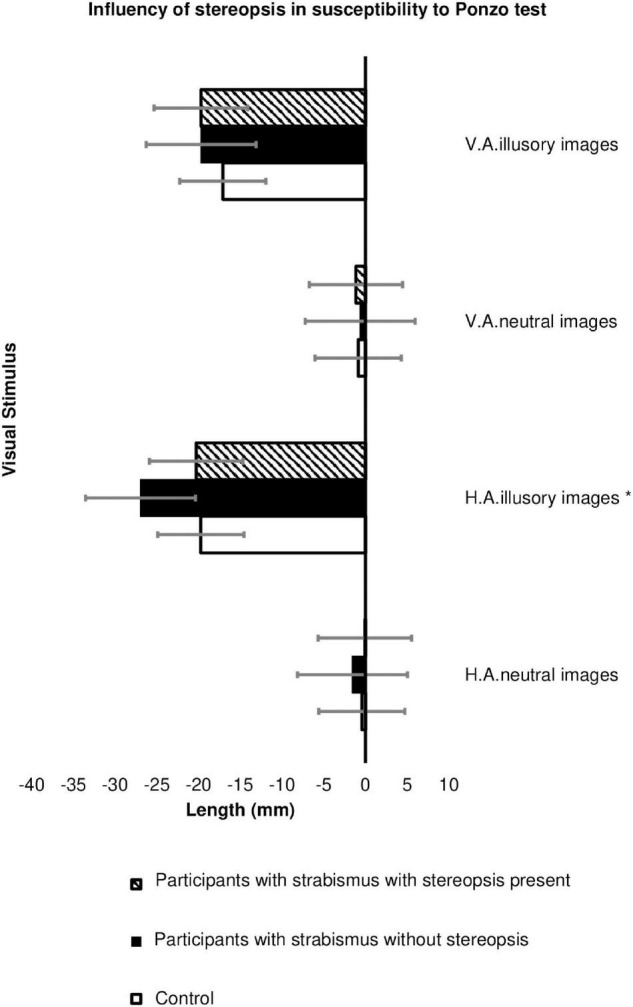
Influence of stereopsis on image size estimation (in millimeters) in the Ponzo test between Groups: Participants with strabismus and preserved stereopsis (*n* = 8), Participants with strabismus without stereopsis (*n* = 37) and Controls (*n* = 62). HA, horizontal adjustment images; VA, vertical adjustment images; Ponzo test: *p-value = 0.007 (Tukey *Post Hoc p* = 0.005) The error bars shown represent the Standard Error.

Only 8 individuals (17.8%) with strabismus had some degree of stereopsis. In Figure 4, it is found that individuals with strabismus that had stereopsis had a similar response to the Control Group regarding the illusory susceptibility in the Ponzo test with horizontal adjustment (−19.72 ± 10.62 mm in the Control Group, versus −20.28 ± 12.41 mm in the Cases group with stereopsis versus −26.95 ± 11.03 mm in the Cases group without stereopsis; *Post Hoc* Tukey *p* = 0.005; Supplementary Tables 4, 5).

**FIGURE 5:**
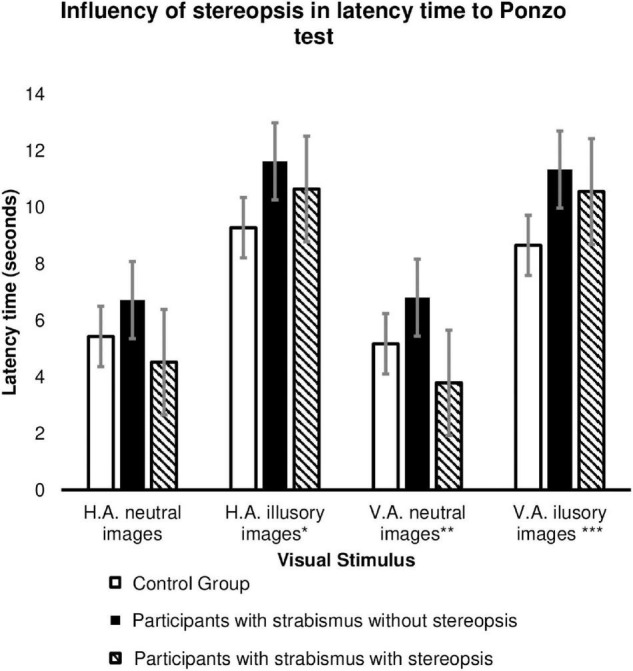
Influence of stereopsis on image size estimation (in millimeters) in the Ponzo test between Groups: Participants with strabismus and preserved stereopsis (*n* = 8), Participants with strabismus without stereopsis (*n* = 37) and Controls (*n* = 62). HA, horizontal adjustment images; VA, vertical adjustment images; HA illusory images *p* value = 0.023* (Tukey *Post-Hoc p* value = 0.018); VA neutral images *p* value = 0.037** (Games-Howeel = 0.022); VA illusory images *p* value = 0.028*** (Turkey *Post-Hoc* = 0.024). The error bars shown represent the Standard Error.

Figure 5 demonstrates that the presence of stereopsis influences the response time for the Ponzo test, which is similar between participants with strabismus who have preserved stereopsis and the Control group. There was a significant slowness in the response time of patients with strabismus without stereopsis for horizontal adjustment illusory images (*p*-value 0.023, Supplementary Table 4; Tukey *Post-Hoc p*-value = 0.018, Supplementary Table 6); vertical adjustment neutral images (*p*-value = 0.037, Supplementary Table 4; Games-Howell = 0.022, Supplementary Table 6); vertical adjustment illusory images (*p*-value = 0.028, Supplementary Table 4; Turkey *Post-Hoc* = 0.024, Supplementary Table 6) compared to patients in the Control Group.

**FIGURE 6:**
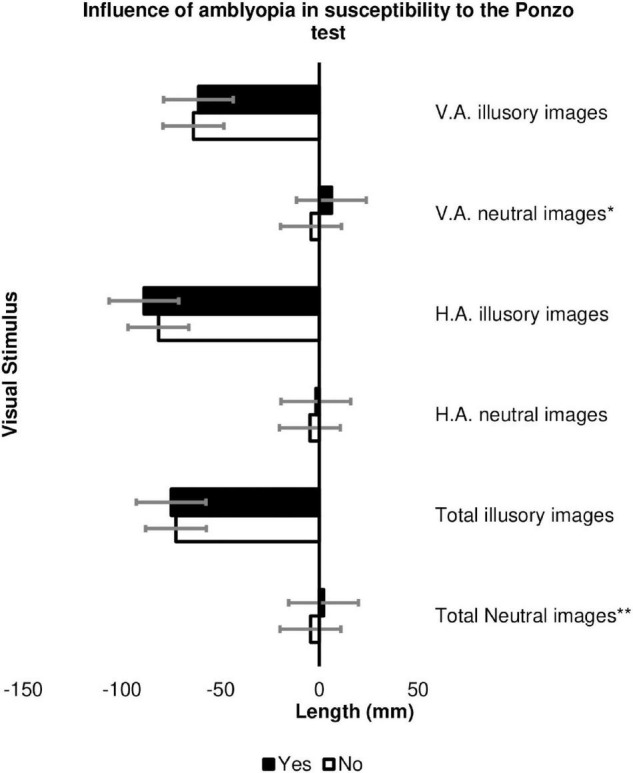
Influence of the presence of amblyopia on the estimation of image size (in millimeters) in the Ponzo test in the Cases Group among patients with strabismus and amblyopia (*n* = 8) and patients with strabismus but without amblyopia (*n* = 37). HA, horizontal adjustment; VA, vertical adjustment; *p-value = 0.005, **p-value = 0.046. The error bars shown represent the Standard Error.

Amblyopia was a significant factor in the estimation of image size in the Ponzo test for neutral images in general (*p* = 0.046), especially those with vertical adjustment (*p* = 0.005), with patients with amblyopia tending to have significant hypercorrection in the estimation of the size of the image (6.17 ± 6.25 mm in the amblyopic versus −4.15 ± 9.28 mm in the non-amblyopic individuals with strabismus) (Figure 6 and Supplementary Table 5).

As for the response time, individuals with strabismus and amblyopia were faster when evaluating the Vertical-horizontal test (*p* = 0.022), especially for vertical adjustment images (11.088 ± 4.972 s in non-amblyopic patients versus 7.902 ± 1.985 s in patients with amblyopia; *p* value = 0.006; Supplementary Table 7).

**FIGURE 7:**
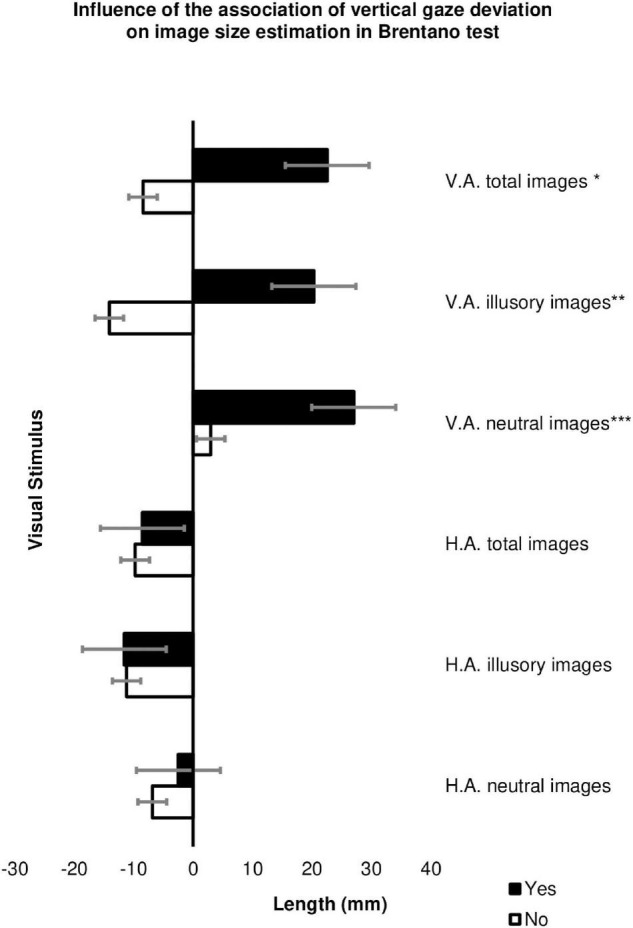
Influence of the presence of vertical strabismus associated with horizontal strabismus to estimate image size (in millimeters) in the Brentano test in the Cases Group among patients with strabismus and associated vertical deviation (*n* = 19) and patients with strabismus without associated vertical deviation (*n* = 29). HA, horizontal adjustment; VA, vertical adjustment; *p-value = 0.017, **p-value = 0.015, ***p-value = 0.016 The error bars shown represent the Standard Error.

Of the 45 participants with strabismus in the study, 26 showed vertical gaze deviation (DVD and/or hypertrophy, and/or alphabetic anisotropies) associated with horizontal strabismus. In these individuals, overcorrection of neutral images with vertical adjustment (*p* = 0.016), illusions with vertical adjustment (*p* = 0.015) and images in general with vertical adjustment (*p* = 0.017) were observed for the Brentano test (Figure 7 and Supplementary Table 8).

For neutral (non-illusory) images, there was a significant difference in the estimate of the size of the image for a vertical adjustment, in the Brentano test (−2.12 ± 38.57 mm in the Control group versus 16.86 ± 33.51 mm in the Cases Group; *p* = 0.009) and the Vertical-Horizontal test (−17.36 ± 36.28 mm in the Control Group versus 7.43 ± 31.73 mm in the Cases Group; *p* = 0.000) and individuals with strabismus tended to overcorrect the length of the adjusted straight line (Supplementary Table 3).

Considering that all individuals in the Cases group had a referral for surgery, only two were diagnosed with small ocular deviation (up to 15Δ), 17 had medium-sized ocular deviation (15–30Δ) and 26 participants had an ocular deviation considered large (greater than 30Δ). There was no significant difference regarding the influence of amount of ocular deviation, strabismus subtypes (converging or divergent) and its time of onset (congenital or infantile deviations) both in relation to image size estimation and in evaluating the response time.

## Discussion

The main aim of this study was to investigate how children/adolescents with strabismus perceive two-dimensional geometric visual illusions in relation to a group of healthy individuals. Despite being classic and widely studied images, these figures had not been evaluated in this population group, so far.

The results obtained showed that individuals with strabismus had greater susceptibility and response time than healthy individuals in the evaluation of Ponzo’s illusory images. Individuals with strabismus and some degree of stereopsis had a similar response to the Control Group when evaluating illusory images. Despite being presented as a two-dimensional figure, Leibowitz et al. (1969) pointed out that the Ponzo illusion presents cues (monocular and binocular) that are normally associated with three-dimensional images of the environment around us, signaling, to the organism, the need to correct the perceived size of the retina for distant objects (Leibowitz et al., 1969). It is argued that the magnitude of the illusory perception results from the “framing effect” that comes from the interaction between the oblique horizontal and vertical lines evoking a sense of three-dimensionality (3D view) (Yamagami, 2007) which could justify the data found. The maximum illusory effect on the layout of the image of the horizontal adjustment found corroborated that reported by Yamagami (2007) in healthy individuals.

Supporting the results found in our study, Cretenoud et al. (2020) demonstrated that individual responses to the magnitude of illusory visual perception of Müller-Lyer and Ponzo are independent of the context in which the image is presented. That is, the susceptibility to a simple version or a three-dimensional version of the Ponzo figure, for the same individual, is strongly correlated. However, although Ponzo and Müller-Lyer are classified as geometric illusions, there is a weak correlation in the magnitude of illusory perception between these two illusions (Cretenoud et al., 2020).

An interesting aspect is that, according to Murray et al. (2006), in a study using Functional Nuclear Magnetic Resonance (fNMR), the brain activity of healthy individuals in area V1 was similar for both visual exposure to a two-dimensional and three-dimensional Ponzo image (Murray et al., 2006). Furthermore, according to Song et al. (2011), Ponzo’s illusory interpretation is mediated by binocular neurons, that is, under conditions of monocular vision, the researchers reported greater susceptibility to the illusion than under binocular conditions which demonstrates complete interocular transfer (mediated by a population of binocular neurons that receive and combine the information from both eyes) (Song et al., 2011). In this regard, a lack of stereopsis and the absence of normal binocular vision represents a great disadvantage for individuals with strabismus in the assessment of the two-dimensional Ponzo illusory images, as these patients use secondary cues for depth perception such as: parallax, shadow, perspective, movement and vanishing point, aspects more susceptible to deception than binocular judgment.

Likewise, it is described in the literature that individuals with strabismus present saccadic movements that do not combine amplitude and direction, in addition to anomalous saccadic velocity, which is worse in individuals without stereopsis and with a greater angle of ocular deviation (Ghasia et al., 2018). Such aspects can compromise the spatial and temporal location of objects (Niechwiej-Szwedo et al., 2017), and has an influence on essential daily activities such as reading, which tends to be slower (Ridha et al., 2014). Although the measurement of eye movements is not part of the object of this study, possibly the slowness in the analysis, interpretation and response of Ponzo’s illusory figures (which demands more complex interpretation) may result from the difficulty in coordinating eye movements experienced by patients with strabismus, where this is even worse in individuals with strabismus without three-dimensional vision (Bucci et al., 2009; Niechwiej-Szwedo et al., 2017; Mihara et al., 2019).

We did not find a similar association for estimating the size of illusory images in the Brentano and Vertical-Horizontal tests. However, Axelrod et al. (2017), studying the cortical response to geometric illusory visual exposure, observed that several areas of the brain were involved in the interpretation of illusory images, which may be analogous to the behavioral response. The authors believe that, despite being classified as geometric illusions, the locus of brain response to interpret these images was not entirely the same, which illustrates the complexity of the visuo-spatial integration (Axelrod et al., 2017). Several studies supported this understanding and advocated that the interpretation of illusory processing at the central level is complex and should not be attributed equally to all geometric illusions, in view of the different forms of presentation of visual stimuli and their context (Cretenoud et al., 2019, 2020; Yildiz et al., 2021).

According to Grave et al. (2006), in healthy individuals, Brentano’s illusory effect is similar in all gaze positions (up, down, right, and left) and in vertical and horizontal adjustment images (Grave et al., 2006). However, when evaluating the influence of the association of vertical component strabismus to horizontal ocular deviation, we observed that the subgroup with a vertical gaze deviation shifted the center of the vertical adjustment axis of the Brentano figure significantly more. The same pattern did not occur in the analysis of the horizontal adjustment images. We could suggest that the difficulty in correctly estimating the size of illusory and neutral vertically adjusted images may be a consequence of the coexistence of vertical and horizontal strabismus, which would make it more difficult to estimate the size of the figures.

The individuals with strabismus showed significant inaccuracy in estimating the length of simple figures, without any visual context, in the neutral images of the Brentano test and the Vertical-Horizontal test. A similar result was also observed in patients with amblyopia who presented a significant difference in the estimation of the size of the vertically adjusted Ponzo neutral images, tending to hypercorrection. Such findings may be related to the difficulty in integrating contours, as described by Niechwiej-Szwedo et al. (2017). The authors also report deficits in tracking and enumeration of objects and changes in sensitivity to movement, findings that reflect high central level involvement (extrastriatum) in addition to changes in primary image processing in patients with amblyopia (Niechwiej-Szwedo et al., 2017).

Another aspect to be considered in the patients with amblyopia is the one raised by Miller (2000), who advocates that the intrinsic property of illusory images allows them to be noticed even if observed in lower definition (as a blurred image), which may justify the similar susceptibility to Ponzo’s illusory images (Miller, 2000). Furthermore, Otero-Millan et al. (2014) demonstrated that binocular performance is superior to monocular performance in the evaluation of illusory figures (Otero-Millan et al., 2014). As such, it could be understood that the binocular presentation of images, without a monocular comparative evaluation, represents a limitation in our study (Ninio, 2014). However, considering the age of the participants in our study, it was decided to perform the tests binocularly to prevent possible influences from external factors, such as tiredness, which could significantly impact performance. Furthermore, we emphasize that our visual perception is routinely carried out binocularly, so the experiment would be more accurate in interpreting reality.

In conclusion, for the first time that we are aware of, it was demonstrated that patients with strabismus present alterations in the perception of geometric illusions and neutral (non-illusory) figures, in a more significant manner for Ponzo’s illusory figures. Thus, we believe that the study of illusory perception constitutes a new approach that can be applied in the visual functional assessment of the individuals with strabismus, in order to help understand the difficulties presented by these patients in their interaction with their surroundings. The response to this simple group of figures may reflect the complex changes in the organization of the Central Nervous System experienced by these patients. Future studies evaluating geometric illusions in association with imaging techniques such as Functional Nuclear Magnetic Resonance may contribute to a better understanding of morpho-functional changes in individuals with strabismus, helping to understand and locate the areas of the brain activated in the presentation of illusory images and the correlation of these findings to those described in terms of behavioral aspects. Illusory images can also serve as a basis for postoperative follow-up in these individuals, improving the understanding of the functional improvements resulting from a surgical procedure for ocular alignment.

## Data Availability Statement

The raw data supporting the conclusions of this article will be made available by the authors, without undue reservation.

## Ethics Statement

The studies involving human participants were reviewed and approved by Research Ethics Committee of the University of Brasília (UnB) (CAAE: 83515717.7.0000.0030). Clinical Trial registered at the Rebec (Brazilian Registry of Clinical Trials) under number RBR-4tzjjc3. Written informed consent to participate in this study was provided by the participants’ legal guardian/next of kin.

## Author Contributions

JR, CI, and MT contributed substantially to the conception and design of the work, essay writing, and critical review of the manuscript. JR, AG, AL, and PS worked on the acquisition, statistical analyses, and interpretation of data for the work. JR, CI, AL, AG, PS, and MT have agreed to be responsible for all aspects of the engagement to ensure that issues relating to the accuracy or completeness of any part of the engagement are properly investigated and resolved and approved the submitted version.

## Conflict of Interest

The authors declare that the research was conducted in the absence of any commercial or financial relationships that could be construed as a potential conflict of interest.

## Publisher’s Note

All claims expressed in this article are solely those of the authors and do not necessarily represent those of their affiliated organizations, or those of the publisher, the editors and the reviewers. Any product that may be evaluated in this article, or claim that may be made by its manufacturer, is not guaranteed or endorsed by the publisher.
