# Supplementary material for: Study of Geometric Illusory Visual Perception – A New Perspective in the Functional Evaluation of Children With Strabismus
Source: Front Hum Neurosci. 2022 Apr 13;16:769412. doi: 10.3389/fnhum.2022.769412 (PMC9043129; doi:10.3389/fnhum.2022.769412)
Supplement: Supplementary file 1 [file Table_1.DOCX]

**Table S1. Clinical characteristics of participants with strabismus.** Key: F, female; M, male; RE, right eye; LE, left eye; ET NA, Esotropia subtype Acquired Nonaccommodative; ET I, Esotropia subtype Infantile; ET A, Esotropia subtype Accommodative; XT, Exotropia subtype Constant; X(T), Exotropia subtype Intermittent; DVD, Dissociated Vertical Deviation; PD, prism diopters; “V”, “V” pattern anisotropy; “A”, “A” pattern anisotropy; N, no associated strabismus; VD, Vertical Deviation.

| **Participant** | **Age (years)** | **Sex** | **Corrected visual acuity (RE)** | **Corrected visual acuity (LE)** | **Refraction (RE)** | **Refraction (LE)** | **Classification of strabismus** | **Vertical Deviation, DVD and/or Anisotropy** | **Strabismus Angle (PD)** | **Stereopsis**  **(Titmus)** |
| --- | --- | --- | --- | --- | --- | --- | --- | --- | --- | --- |
| 1 | 12 | F | 6/10 | 5/10 | +1.00 | +1.00 | ET NA | "A" | ET 30 | ZERO |
| 2 | 13 | M | 8/10 | 8/10 | -1.00 | -1.00 | X(T) | N | X(T) 16 | 400" |
| 3 | 14 | F | 10/10 | 10/10 | +5.00 (-0.50) 90° | +4.25 (-0.25) 90° | ET A | N | ET 65 | ZERO |
| 4 | 15 | M | 10/10 | 10/10 | +0.75 (-0.50) 180° | +0.75 (-0.50) 180° | ET NA | VD | ET 25+10 | ZERO |
| 5 | 13 | M | 10/10 | 10/10 | +1.50 (-0.50) 180° | +1.25 (-0.50) 180° | ET I | "V" | ET 40 | ZERO |
| 6 | 11 | M | 10/10 | 10/10 | +0.25 (-0.75) 10° | 0.00 (-0.50) 180° | ET NA | "V" | ET 40 | 140" |
| 7 | 12 | F | 10/10 | 10/10 | +1.00 (-0.75) 180° | +1.00 (-0.75) 180° | ET I | DVD | ET 50 | ZERO |
| 8 | 13 | F | 10/10 | 10/10 | +0.75 | +1.00 | ET I | DVD / VD | ET 14+12 | ZERO |
| 9 | 10 | M | 10/10 | 10/10 | -0.75 (-0.25) 30° | -1.25 (-0.50) 170° | ET I | VD | ET 35+5 | ZERO |
| 10 | 11 | F | 10/10 | 10/10 | -2.50 (-0.50) 180° | -2.50 (-0.25) 165° | X(T) | N | X(T)30 | 40" |
| 11 | 12 | F | 10/10 | 10/10 | +1.00 (-0.75) 170° | -1.00 (-0.50) 180° | ET I | VD | ET 12+14 | ZERO |
| 12 | 13 | M | 10/10 | 10/10 | 0.00 (-1.00) 120° | 0.00 (-1.00) 120° | ET I | DVD | ET 40 | ZERO |
| 13 | 10 | F | 10/10 | 10/10 | 0.00 (-1.00) 120° | 0.00 (-1.00) 120° | ET I | VD | ET 50-8 | ZERO |
| 14 | 11 | F | 5/10 | 10/10 | +1.50 (-1.00) 10° | +1.75 (-0.25) 140° | ET A | DVD | ET 45 | ZERO |
| 15 | 13 | M | 10/10 | 10/10 | +1.25 (-0.75) 180° | +1.25 (-0.75) 165° | X(T) | N | X(T) 25 | 40" |
| 16 | 10 | M | 10/10 | 10/10 | -0.25 (-0.50) 120° | -0.50 (-0.50) 60° | ET I | "V" | ET 30 | ZERO |
| 17 | 10 | M | 8/10 | 8/10 | +6.00 (-2.00) 10° | +6.00 (-2.25) 175° | ET A | N | ET 50 | ZERO |
| 18 | 10 | F | 10/10 | 6/10 | +5.75 (-0.75) 170° | +7.00 (-2.00) 15° | ET A | VD | ET 50+8 | ZERO |
| 19 | 13 | M | 5/10 | 6/10 | +0.50 (-2.50) 20° | +0.75 (-2.25) 160° | XT | N | XT 23 | ZERO |
| 20 | 11 | M | 10/10 | 10/10 | +1.00 (-2.00) 5° | +2.00 (-2.50) 170° | ET I | N | ET 35 | ZERO |
| 21 | 10 | M | 10/10 | 10/10 | +2.00 (-0.25) 180° | +2.50 (-0.75) 180° | ET I | "V" | ET 40-6 | ZERO |
| **Continue** |  |  |  |  |  |  |  |  |  |  |
| 22 | 10 | F | 10/10 | 10/10 | +6.75 (-0.75) 175° | +6.75 (-0.50) 180° | ET A | DVD | ET 45 | ZERO |
| 23 | 15 | F | 10/10 | 10/10 | 0.00 | 0.00 | XT | "V" | XT 40-4 | ZERO |
| 24 | 13 | F | 8/10 | 10/10 | +1.00 (-1.50) 5° | +1.50 (-1.50) 180° | ET I | "V" | ET 45+2 | ZERO |
| 25 | 11 | M | 10/10 | 10/10 | -0,75 (-0,50) 165° | -0.75 | ET I | "A" | ET 50-5 | ZERO |
| 26 | 11 | M | 8/10 | 4/10 | +8.50 (-3.50) 175° | +7.75 (-3.50) 5° | ET A | "V" | ET 50 | ZERO |
| 27 | 10 | M | 10/10 | 10/10 | +1.00 | +1.00 | ET NA | "A" | ET 35 | ZERO |
| 28 | 10 | M | 10/10 | 10/10 | -0.25 (-1.25) 85° | -0.50 (-0.50) 75° | X(T) | "V" | XT 18+4 | ZERO |
| 29 | 10 | F | 8/10 | 8/10 | -2.00 (-2.75) 165° | -3.00 (-2.50) 180° | XT | "A" | XT 25+3 | ZERO |
| 30 | 13 | M | <2/10 | 5/10 | +1.25 (-1.25) 130° | +1.25 (-1.25) 180° | ET I | N | ET 35 | ZERO |
| 31 | 11 | F | 10/10 | 6/10 | +0.50 (-0.50) 180 ° | +1.00 (-0.25) 180° | ET I | DVD | ET 30 | ZERO |
| 32 | 12 | M | 10/10 | 10/10 | +2.50 | +2.75 (-1.00) 20° | ET I | "V" | ET 50+6 | ZERO |
| 33 | 12 | M | 10/10 | 10/10 | +2.50 | +2.50 | ET NA | N | ET 60 | ZERO |
| 34 | 15 | M | 10/10 | 10/10 | 0.00 (-0.50) 180° | 0.00 (-0.50) 160° | X(T) | N | X(T) 25 | 40" |
| 35 | 13 | M | 10/10 | 10/10 | +0.25 (-0.75) 165° | +0.25 (-0.75) 180° | X(T) | N | X(T) 20 | 140" |
| 36 | 15 | F | 10/10 | 10/10 | 0.00 | 0.00 | X(T) | N | XT 50 | 400" |
| 37 | 14 | M | 10/10 | 10/10 | +1.00 (-1.00) 175° | +1.00 (-1.00) 180° | ET NA | DVD | ET 30 | ZERO |
| 38 | 10 | M | 5/10 | 6/10 | +4.25 (-3.50) 180° | +3.00 (-2.25) 170° | ET I | N | ET45 | ZERO |
| 39 | 15 | F | 8/10 | 2/10 | -5.50 (-2.50) 115° | -10.50 | ET NA | N | ET 50 | ZERO |
| 40 | 14 | F | 10/10 | 2/10 | +0.75 (-0.50) 180° | +1.50 (-0.75) 10° | ET I | "V" | ET 45 | ZERO |
| 41 | 11 | F | 10/10 | 10/10 | +0.50 (-1.00) 180° | 0.00 (-0.50) 180° | X(T) | "V" | XT 40 | ZERO |
| 42 | 11 | M | 10/10 | 10/10 | -0.75 (-0.50) 175° | -1.00 (-0.50) 165° | XT | N | XT 45 | ZERO |
| 43 | 12 | M | 10/10 | 10/10 | +4.25 (-1.25) 165° | +3.50 (-1.00) 180° | ET A | "V" | ET35+10 | ZERO |
| 44 | 11 | M | 10/10 | 10/10 | 0.00 (-0.50) 180° | 0.00 (-0.50) 180° | X(T) | N | XT 20 | 140" |
| 45 | 12 | M | 8/10 | 8/10 | -1.00 (-0.75) 140° | -1.00 (-0.50) 45° | ET I | "A" | ET 50 | ZERO |
