# Supplementary material for: Study of Geometric Illusory Visual Perception – A New Perspective in the Functional Evaluation of Children With Strabismus
Source: Front Hum Neurosci. 2022 Apr 13;16:769412. doi: 10.3389/fnhum.2022.769412 (PMC9043129; doi:10.3389/fnhum.2022.769412)
Supplement: Supplementary file 2 [file Table_2.DOCX]

**Table S2: Physical specifications of the visual stimuli presented**. Key: * total number of neutral images per session, † total number of illusory images per session.

| **Visual Stimuli** | **Neutral images** | **Illusionary images** |
| --- | --- | --- |
| Vertical-horizontal (4*,8†) | Stroke width = 2 pixels = 1.5 mm  Length of straight lines = 48 mm  Angle between segments = 90º | Stroke width = 2 pixels = 1.5 mm  Length of straight lines = 48 mm approx. 6º of visual angle  Angle between segments = 90º |
| Muller-Lyer (Brentano version) (12*, 24†) | Stroke width = 2 pixels = 1.5 mm  Length of the longest straight = 105.8 mm  Center of the longest straight line (point of subjective equality) = 52.90 mm  Length of perpendicular lines = 18.0 mm  Angle of perpendicular lines = 90º in relation to the longest line  Shift from the central line = 18.66 mm | Stroke width = 2 pixels = 1.5 mm  Length of the longest straight line = 105.8 mm approx. 12º visual angle  Center of the longest straight line (point of subjective equality) = 52.90 mm  Length of fins = 18.0 mm  Angle of the fins = 30º in relation to the longest straight line  Shift of the central fin = 18.66 mm |
| Pozo (8*^,^ 8†) | Stroke width = 2 pixels = 1.5 mm  Length of parallel lines = 24 mm  Separation between parallel lines = 60mm | Stroke width = 2 pixels = 1.5 mm  Figure height = 114.0 mm approx. 12º visual angle  Length of parallel lines = 24 mm  Separation between parallel lines = 60mm  Shortest distance between oblique lines = 27.5 mm  Longest distance between oblique lines = 96.3 mm  Angle of inclination of oblique lines = 20º |
