# Supplementary material for: Study of Geometric Illusory Visual Perception – A New Perspective in the Functional Evaluation of Children With Strabismus
Source: Front Hum Neurosci. 2022 Apr 13;16:769412. doi: 10.3389/fnhum.2022.769412 (PMC9043129; doi:10.3389/fnhum.2022.769412)
Supplement: Supplementary file 3 [file Table_3.DOCX]

**Table S3. Estimate of the image size (in millimeters) and Response time (in seconds) to adjust the image between patients with Strabismus versus Control Group.** Key: *Degrees of Freedom = 105; *Diff,* Difference in measuring image size (in milimeters); *Δ t,* Latency time to adjust the image (in seconds); M, mean; SD, standart deviation; Med, Median.

|  |  |  | **Control Group**  **(n 62)** | | | **Strabismic patients**  **(n 45)** | | | **Test T *** | |
| --- | --- | --- | --- | --- | --- | --- | --- | --- | --- | --- |
| **Ajustament images presented** | **Test** |  | **M** | **SD** | **Med** | **M** | **SD** | **Med** | **t** | **p-value** |
| Neutral images | Vertical-Horizontal | *Diff* | -5.520 | 19.177 | 0.000 | 6.810 | 24.398 | 0.000 | -2.926 | **.004** |
|  |  | *Δ t* | 8.721 | 5.870 | 7.559 | 7.797 | 5.372 | 6.640 | .833 | .407 |
|  | Brentano | *Diff* | 0.295 | 27.094 | 1.221 | 6.264 | 26.351 | 7.317 | -1.138 | .258 |
|  |  | *Δ t* | 7.894 | 4.342 | 7.144 | 7.634 | 2.628 | 7.549 | .357 | .722 |
|  | Ponzo | *Diff* | -2.092 | 10.768 | -1.369 | -3.241 | 8.617 | -0.456 | .591 | .556 |
|  |  | *Δ t* | 5.306 | 2.556 | 5.325 | 6.305 | 3.499 | 5.535 | -1.706 | .091 |
| Illusory images | Vertical-Horizontal | *Diff* | -129.249 | 114.222 | -118.000 | -153.237 | 105.128 | -137.263 | 1.108 | .270 |
|  |  | *Δ t* | 11.296 | 5.825 | 10.239 | 11.301 | 4.062 | 10.399 | -.005 | .996 |
|  | Brentano | *Diff* | -5.352 | 39.812 | 0.156 | -2.816 | 34.101 | -1.821 | -.345 | .731 |
|  |  | *Δ t* | 9.178 | 4.410 | 8.205 | 9.158 | 3.027 | 8.784 | .026 | .979 |
|  | Ponzo | *Diff* | -59.153 | 33.875 | -57.188 | -73.063 | 29.687 | -67.700 | 2.207 | **.030** |
|  |  | *Δ t* | 8.974 | 3.323 | 8.566 | 11.336 | 5.067 | 10.688 | -2.911 | **.004** |
| Total images | Vertical-Horizontal | *Diff* | -88.006 | 76.813 | -78.083 | -99.888 | 69.994 | -92.733 | .820 | .414 |
|  |  | *Δ t* | 10.438 | 5.199 | 8.838 | 10.133 | 3.999 | 9.346 | .329 | .746 |
|  | Brentano | *Diff* | -3.470 | 31.485 | 1.729 | 0.211 | 29.156 | 1.022 | -.616 | .540 |
|  |  | *Δ t* | 8.750 | 4.278 | 7.919 | 8.650 | 2.824 | 8.575 | .136 | .892 |
|  | Ponzo | *Diff* | -30.623 | 19.655 | -28.591 | -38.152 | 15.964 | -33.856 | 2.112 | **.037** |
|  |  | *Δ t* | 7.140 | 2.572 | 6.794 | 8.820 | 3.533 | 8.556 | -2.849 | **.005** |
| Horizontal Adjustment neutral images | Vertical-Horizontal | *Diff* | 6.326 | 24.734 | 0.000 | 6.180 | 29.207 | 0.000 | .028 | .978 |
|  |  | *Δ t* | 8.118 | 5.841 | 6.957 | 7.204 | 4.824 | 5.830 | .858 | .393 |
|  | Brentano | *Diff* | 2.718 | 52.041 | 1.217 | -4.335 | 38.607 | -3.650 | .768 | .444 |
|  |  | *Δ t* | 7.875 | 4.540 | 7.330 | 7.314 | 2.869 | 6.901 | .729 | .468 |
|  | Ponzo | *Diff* | -1.370 | 10.201 | 0.000 | -4.164 | 13.671 | -1.825 | 1.211 | .229 |
|  |  | *Δ t* | 5.435 | 3.108 | 5.153 | 6.334 | 3.748 | 5.693 | -1.354 | .179 |
| Continue |  |  |  |  |  |  |  |  |  |  |
| Horizontal Adjustment illusory images | Vertical-Horizontal | *Diff* | -85.024 | 111.344 | -62.175 | -102.796 | 107.445 | -76.850 | .827 | .410 |
|  |  | *Δ t* | 11.205 | 8.481 | 9.354 | 11.013 | 3.928 | 10.551 | .141 | .888 |
|  | Brentano | *Diff* | -11.010 | 56.729 | -5.188 | -11.402 | 48.465 | -13.425 | .037 | .970 |
|  |  | *Δ t* | 9.429 | 4.897 | 8.156 | 9.302 | 3.153 | 9.021 | .153 | .876 |
|  | Ponzo | *Diff* | -63.385 | 34.143 | -65.875 | -82.824 | 36.750 | -78.700 | 2.815 | **.006** |
|  |  | *Δ t* | 9.287 | 3.521 | 8.510 | 11.460 | 4.697 | 11.168 | -2.737 | **.007** |
| Horizontal Adjustment images | Vertical-Horizontal | *Diff* | -54.574 | 76.943 | -36.600 | -66.471 | 71.829 | -51.267 | .812 | .419 |
|  |  | *Δ t* | 10.176 | 6.478 | 8.400 | 9.743 | 3.589 | 9.229 | .405 | .686 |
|  | Brentano | *Diff* | -6.434 | 47.554 | -5.897 | -9.047 | 40.428 | -10.161 | .298 | .766 |
|  |  | *Δ t* | 8.911 | 4.570 | 8.025 | 8.639 | 2.897 | 8.432 | .351 | .726 |
|  | Ponzo | *Diff* | -32.378 | 19.428 | -33.563 | -43.494 | 20.663 | -41.175 | 2.845 | **.005** |
|  |  | *Δ t* | 7.361 | 2.744 | 6.878 | 8.897 | 3.721 | 8.646 | -2460 | **.016** |
| Vertical Adjustment neutral images | Vertical-Horizontal | *Diff* | -17.365 | 36.286 | -5.475 | 7.439 | 31.739 | 0.000 | -3.676 | **.000** |
|  |  | *Δ t* | 9.325 | 7.259 | 7.907 | 8.391 | 6.774 | 6.989 | .675 | .501 |
|  | Brentano | *Diff* | -2.129 | 38.570 | 1.217 | 16.862 | 33.516 | 13.417 | -2.654 | **.009** |
|  |  | *Δ t* | 7.913 | 4.351 | 6.708 | 7.954 | 2.793 | 8.002 | -.056 | .956 |
|  | Ponzo | *Diff* | -2.814 | 14.487 | 0.000 | -2.318 | 9.631 | 0.000 | -.200 | .842 |
|  |  | *Δ t* | 5.178 | 2.895 | 4.446 | 6.275 | 5.132 | 4.936 | -1.405 | .163 |
| Vertical Adjustment illusory images | Vertical-Horizontal | *Diff* | -173.474 | 129.516 | -169.025 | -203.678 | 116.718 | -177.525 | 1.241 | .217 |
|  |  | *Δ t* | 11.386 | 5.046 | 10.429 | 11.588 | 4.663 | 10.388 | -.211 | .833 |
|  | Brentano | *Diff* | 0.306 | 50.775 | 3.958 | 5.771 | 47.749 | -1.825 | -.563 | .574 |
|  |  | *Δ t* | 8,927 | 4.517 | 8.146 | 9.014 | 3.043 | 8.716 | -.113 | .911 |
|  | Ponzo | *Diff* | -54.921 | 39.377 | -53.988 | -63.301 | 30.958 | -57.663 | 1.186 | .238 |
|  |  | *Δ t* | 8.661 | 3.793 | 7.823 | 11.212 | 5.990 | 9.712 | -2.693 | **.008** |
| Vertical Adjustment images | Vertical-Horizontal | *Diff* | -121.438 | 87.709 | -125.058 | -133.306 | 78.525 | -118.350 | .722 | .472 |
|  |  | *Δ t* | 10.699 | 5.178 | 9.683 | 10.522 | 4.730 | 10.080 | .181 | .857 |
|  | Brentano | *Diff* | -0.505 | 40,.422 | 1.831 | 9.468 | 39.045 | 10.172 | -1.278 | .204 |
|  |  | *Δ t* | 8.589 | 4.235 | 7.744 | 8.661 | 2.840 | 8.471 | -.099 | .921 |
|  | Ponzo | *Diff* | -28.868 | 22.940 | -26.081 | -32.810 | 16.624 | -28.363 | .980 | .329 |
|  |  | *Δ t* | 6.919 | 2.819 | 6.473 | 8.744 | 4.116 | 8.063 | -2.721 | **.008** |
