# Supplementary material for: Study of Geometric Illusory Visual Perception – A New Perspective in the Functional Evaluation of Children With Strabismus
Source: Front Hum Neurosci. 2022 Apr 13;16:769412. doi: 10.3389/fnhum.2022.769412 (PMC9043129; doi:10.3389/fnhum.2022.769412)
Supplement: Supplementary file 4 [file Table_4.DOCX]

**Table S4. Influence of stereopsis on image size estimation (mm) and response time (in seconds) between the Groups: Control, Strabismic patients with preserved Stereopsis and Strabismic Patients without preserved Stereopsis.** Key: *Diff,* Difference in measuring image size (in millimeters); *Δ t,* Latency time to adjust the image (in seconds); M mean; SD, standard deviation; CI, Confidence Interval; *Degrees of Freedom (Between-Subjects = 2, Within-Subjects = 104, Total = 106); † Welch.

|  |  |  | **Stereopsis** | | |  |  | |
| --- | --- | --- | --- | --- | --- | --- | --- | --- |
|  |  |  | **Control group**  **(n 62)** | **Strabismic Patients (n 45)** | |  |  | |
| **Adjustment images presented** | **Test** |  |  | **No**  **(n 37)** | **Yes**  **(n 8)** |  | **One Way**  **ANOVA** | |
|  |  |  | **M (SD)** | **M (SD)** | **M (SD)** | **Levene Test *** | **F** | **p-value** |
| Neutral images | Vertical-Horizontal | *Diff* | -1.717 (5.966) | 1.546 (7.887) | 4.766 (5.702) | .300 | 5.065 | **.008** |
|  |  | *Δ t* | 8.721 (5.870) | 8.136 (5.411) | 6.323 (5.236) | .988 | .716 | .491 |
|  | Brentano | *Diff* | .09 (8.43) | 2.41 (8.64) | -.17 (5.70) | .628 | .957 | .387 |
|  |  | *Δ t* | 7.894 (4.342) | 7.865 (2.722) | 6.568 (1.928) | .588 | .462 | .631 |
|  | Ponzo | *Diff* | -.651 (3.350) | -1.096 (2.753) | -.604 (2.441) | .854 | .256 | .775 |
|  |  | *Δ t* | 5.306 (2.556) | 6.768 (3.623) | 4.162 (1.752) | .062 | 4.116 | **.019** |
| Illusory images | Vertical-Horizontal | *Diff* | -40.211(35.536) | -48.409 (29.904) | -44.270 (45.923) | .352 | .656 | .521 |
|  |  | *Δ t* | 11.296 (5.825) | 11.526 (4.145) | 10.259 (3.726) | .572 | .197 | .821 |
|  | Brentano | *Diff* | -1.665 (12.386) | .779 (10.229) | -8.531 (9.375) | .972 | 2.219 | .114 |
|  |  | *Δ t* | 9.178 (4.410) | 9.491 (3.059) | 7.620 (2.488) | .612 | .765 | .468 |
|  | Ponzo | *Diff* | -18.403(10.539) | -23.321 (9.330) | -19.998 (8.840) | .542 | 2.790 | .066 |
|  |  | *Δ t* | 8.974 (3.323) | 11.492 (4.577) | 10.614 (7.273) | .101 | 4.355 | **.015** |
| Total images | Vertical-Horizontal | *Diff* | -27.380 (23.897) | -31.758 (19.701) | -27.925 (31.157) | .284 | .424 | .656 |
|  |  | *Δ t* | 10.438 (5.199) | 10.396 (4.115) | 8.916 (3.374) | .474 | .374 | .689 |
|  | Brentano | *Diff* | -1.079 (9.795) | 1.321 (8.959) | -5.743 (7.580) | .975 | 2.061 | .132 |
|  |  | *Δ t* | 8.750 (4.278) | 8.949 (2.876) | 7.269 (2.224) | .689 | .675 | .511 |
|  | Ponzo | *Diff* | -9.527 (6.115) | -12.209 (5.011) | -10.301 (4.746) | .578 | 2.598 | .079 |
|  |  | *Δ t* | 7.140 (2.572) | 9.130 (3.488) | 7.388 (3.609) | .382 | 5.218 | **.007** |
| Horizontal Adjustment neutral images | Vertical-Horizontal | *Diff* | 1.968 (7.695) | 1.138 (9.117) | 5.549 (8.555) | .442 | .935 | .396 |
|  |  | *Δ t* | 8.118 (5.849) | 7.550 (4.822) | 5.602 (4.807) | .654 | .788 | .458 |
|  | Brentano | *Diff* | .846 (16.191) | -.779 (12.660) | -3.985 (8.538) | .392 | .451 | .638 |
|  |  | *Δ t* | 7.875 (4.540) | 7.573 (3.005) | 6.119 (1.821) | .569 | .716 | .491 |
|  | Ponzo | *Diff* | -.426 (3.174) | -1.561 (4.411) | -.070 (3.396) | .174 | 1.279 | .283 |
|  |  | *Δ t* | 5.435 (3.108) | 6.724 (3.917) | 4.530 (2.212) | .052 | 2.333 | .102 |
|  |  |  |  |  |  |  |  |  |
|  |  |  |  |  |  |  |  |  |
| Continue |  |  |  |  |  |  |  |  |
| Horizontal Adjustment illusory images | Vertical-Horizontal | *Diff* | -26.452 (34.640) | -32.786 (30.298) | -28.258 (47.786) | .305 | .397 | .674 |
|  |  | *Δ t* | 11.205 (8.481) | 11.071 (3.851) | 10.748 (4.540) | .401 | .017 | .983 |
|  | Brentano | *Diff* | -3.425 (17.649) | -1.764 (15.156) | -11.796 (12.374) | .876 | 1.215 | .301 |
|  |  | *Δ t* | 9.429 (4.897) | 9.612 (3.126) | 7.867 (3.063) | .516 | .566 | .570 |
|  | Ponzo | *Diff* | -19.720 (10.622) | -26.953 (11.030) | -20.283 (12.418) | .624 | 5.251 | **.007** |
|  |  | *Δ t* | 9.287 (3.521) | 11.634 (4.509) | 10.659 (5.763) | .248 | 3.913 | **.023** |
| Horizontal Adjustment images | Vertical-Horizontal | *Diff* | -16.979 (23.938) | -21.478 (20.120) | -16.989 (32.220) | .296 | .448 | .640 |
|  |  | *Δ t* | 10.176 (6.478) | 9.897 (3.503) | 9.033 (4.143) | .247 | .163 | .850 |
|  | Brentano | *Diff* | -2.002 (14.795) | -1.435 (12.726) | -9.192 (10.246) | .875 | 1.078 | .344 |
|  |  | *Δ t* | 8.911 (4.570) | 8.932 (2.914) | 7.285 (2.557) | .541 | .633 | .533 |
|  | Ponzo | *Diff* | -10.073 (6.044) | -14.257 (6.098) | -10.177 (7.271) | .796 | 5.565 | **.005** |
|  |  | *Δ t* | 7.361 (2.744) | 9.179 (3.835) | 7.594 (3.008) | .256 | 3.859 | **.024** |
| Vertical Adjustment neutral images | Vertical-Horizontal | *Diff* | -5.402 (11.289) | 1.954 (10.562) | 3.982 (5.937) | .143 | 6.825 | **.002** |
|  |  | *Δ t* | 9.325 (7.259) | 8.722 (6.982) | 6.862 (5.868) | .929 | .454 | .636 |
|  | Brentano | *Diff* | -.662 (12.000) | 5.590 (10.332) | 3.654 (11.442) | .865 | 3.590 | **.031** |
|  |  | *Δ t* | 7.913 (4.351) | 8.157 (2.830) | 7.017 (2.576) | .645 | .300 | .742 |
|  | Ponzo | *Diff* | -.875 (4.507) | -.631 (2.972) | -1.138 (3.279) | .945 | .074 | .929 |
|  |  | *Δ t* | 5.178 (2.895) | 6.812 (5.473) | 3.794 (1.708) | .038 | 17.764† | **.037** |
| Vertical Adjustment illusory images | Vertical-Horizontal | *Diff* | -53.969 (40.294) | -64.033 (34.834) | -60.283 (45.105) | .771 | .793 | .455 |
|  |  | *Δ t* | 11.386 (5.046) | 11.981 (4.839) | 9.769 (3.410) | .580 | .698 | .500 |
|  | Brentano | *Diff* | .095 (15.797) | 3.323 (14.691) | -5.267 (14.415) | .755 | 1.193 | .308 |
|  |  | *Δ t* | 8.927 (4.517) | 9.369 (3.136) | 7.372 (1.966) | .496 | .845 | .432 |
|  | Ponzo | *Diff* | -17.087 (12.250) | -19.690 (10.014) | -19.712 (8.207) | .308 | .696 | .501 |
|  |  | *Δ t* | 8.661 (3.793) | 11.351 (5.246) | 10.569 (9.132) | .064 | 3.683 | **.028** |
| Vertical Adjustment images | Vertical-Horizontal | *Diff* | -37.780 (27.287) | -42.037 (23.342) | -38.862 (30.658) | .703 | .306 | .737 |
|  |  | *Δ t* | 10.699 (5.178) | 10.895 (5.002) | 8.800 (2.808) | .392 | .595 | .553 |
|  | Brentano | *Diff* | -.157 (12.576) | 4.078 (11.836) | -2.294 (12.995) | .876 | 1.697 | .188 |
|  |  | *Δ t* | 8.589 (4.235) | 8.965 (2.924) | 7.254 (1.996) | .598 | .705 | .469 |
|  | Ponzo | *Diff* | -8.981 (7.137) | -10.160 (5.487) | -10.425 (3.640) | .303 | .482 | .619 |
|  |  | *Δ t* | 6.919 (2.819) | 9.081 (4.026) | 7.182 (4.444) | .249 | 4.762 | **.010** |
