# Supplementary material for: Study of Geometric Illusory Visual Perception – A New Perspective in the Functional Evaluation of Children With Strabismus
Source: Front Hum Neurosci. 2022 Apr 13;16:769412. doi: 10.3389/fnhum.2022.769412 (PMC9043129; doi:10.3389/fnhum.2022.769412)
Supplement: Supplementary file 5 [file Table_5.DOCX]

**Table S5.** **Tukey Post-Hoc Test for influence of stereopsis on image size estimation (mm) between the Groups: Control, Strabismic patients with preserved Stereopsis and Strabismic Patients without preserved Stereopsis.**

| Adjustment images presented | Group | Group | Average Difference | Error | p-value | CI 95% | |
| --- | --- | --- | --- | --- | --- | --- | --- |
|  |  |  |  |  |  | Inferior  Limite | Superior  Limite |
| ***Vertical-Horizontal Test***  ***Neutral images*** | Control | Without Stereopsis | -3.263 | 1.387 | .053 | -6.562 | .035 |
|  |  | With Stereopsis | -6.483 | 2.508 | **.030** | -12.448 | -.517 |
|  | Without Stereopsis | Control | 3.263 | 1.387 | .053 | -.0353 | 6.562 |
|  |  | With Stereopsis | -3.219 | 2.603 | .435 | -9.410 | 2.971 |
|  | With Stereopsis | Control | 6.483 | 2.508 | **.030** | .517 | 12.448 |
|  |  | Without Stereopsis | 3.219 | 2.603 | .435 | -2.971 | 9.410 |
| ***Vertical-Horizontal Test***  ***Vertical Adjustment neutral images*** | Control | Without Stereopsis | -7.356 | 2.234 | **.004** | -12.670 | -2.042 |
|  |  | With Stereopsis | -9.384 | 4.041 | .057 | -18.994 | .225 |
|  | Without Stereopsis | Control | 7.356 | 2.234 | **.004** | 2.042 | 12.670 |
|  |  | With Stereopsis | -2.028 | 4.194 | .879 | -12.002 | 7.945 |
|  | With Stereopsis | Control | 9.384 | 4.041 | .057 | -.225 | 18.994 |
|  |  | Without Stereopsis | 2.028 | 4.194 | .879 | -7.945 | 12.002 |
| ***Brentano Test***  ***Vertical Adjustment neutral images*** | Control | Without Stereopsis | -6.252 | 2.370 | **.026** | -11.889 | -.616 |
|  |  | With Stereopsis | -4.315 | 4.286 | .574 | -14.508 | 5.877 |
|  | Without Stereopsis | Control | 6.252 | 2.370 | **.026** | .616 | 11.889 |
|  |  | With Stereopsis | 1.936 | 4.449 | .901 | -8.642 | 12.516 |
|  | With stereopsis | Control | 4.315 | 4.286 | .574 | -5.877 | 14.508 |
|  |  | Without Stereopsis | -1.936 | 4.449 | .901 | -12.516 | 8.642 |
| Continue |  |  |  |  |  |  |  |
| ***Ponzo Test***  ***Horizontal Adjustment illusory images*** | Control | Without Stereopsis | 7.233 | 2.263 | **.005** | 1.852 | 12.614 |
|  |  | With Stereopsis | .563 | 4.092 | .990 | -9.166 | 10.294 |
|  | Without Stereopsis | Control | -7.233 | 2.263 | **.005** | -12.614 | -1.852 |
|  |  | With Stereopsis | -6.669 | 4.247 | .263 | -16.768 | 3.429 |
|  | With Stereopsis | Control | -.563 | 4.092 | .990 | -10.294 | 9,166 |
|  |  | Without Stereopsis | 6.669 | 4.247 | .263 | -3.429 | 16.768 |
| ***Ponzo Test***  ***Horizontal Adjustment images*** | Control | Without Stereopsis | 4.183 | 1.278 | **.004** | 1.144 | 7.223 |
|  |  | With Stereopsis | .103 | 2.311 | .999 | -5.392 | 5.599 |
|  | Without Stereopsis | Control | -4.183 | 1.278 | **.004** | -7.223 | -1,144 |
|  |  | With Stereopsis | -4.080 | 2.399 | .210 | -9.784 | 1.624 |
|  | With Stereopsis | Control | -.103 | 2.311 | .999 | -5.599 | 5,392 |
|  |  | Without Stereopsis | 4.080 | 2.399 | .210 | -1.624 | 9.784 |
