# Supplementary material for: Study of Geometric Illusory Visual Perception – A New Perspective in the Functional Evaluation of Children With Strabismus
Source: Front Hum Neurosci. 2022 Apr 13;16:769412. doi: 10.3389/fnhum.2022.769412 (PMC9043129; doi:10.3389/fnhum.2022.769412)
Supplement: Supplementary file 6 [file Table_6.DOCX]

**Table S6.** **Post-Hoc Test for influence of stereopsis on response time (in seconds) between the Groups: Control, Strabismic patients with preserved Stereopsis and Strabismic Patients without preserved Stereopsis.**

| **Adjustment**  **images presented**  ***Ponzo Test*** | **Statistic**  **Test** | **Group** | **Group** | **Average Difference** | **Error** | **p-value** | CI 95% | |
| --- | --- | --- | --- | --- | --- | --- | --- | --- |
|  |  |  |  |  |  |  | Inferior  **Limite** | Superior  **Limite** |
| ***Neutral images*** | Tukey HSD | Control | Without Stereopsis | -1.461 | .608 | **.047** | -2.908 | -.014 |
|  |  |  | With Stereopsis | 1.144 | 1.100 | .554 | -1.472 | 3.760 |
|  |  | Without Stereopsis | Control | 1.461 | .608 | **.047** | .014 | 2.908 |
|  |  |  | With Stereopsis | 2.605 | 1.142 | .063 | -.110 | 5.321 |
|  |  | With Stereopsis | Control | -1.144 | 1.100 | .554 | -3.760 | 1.472 |
|  |  |  | Without Stereopsis | -2.605 | 1.142 | .063 | -5.321 | .110 |
| ***Illusory images*** | Tukey HSD | Control | Without Stereopsis | -2.518 | .863 | **.012** | -4.572 | -.464 |
|  |  |  | With Stereopsis | -1.640 | 1.562 | .547 | -5.354 | 2.074 |
|  |  | Without Stereopsis | Control | 2.518 | .863 | .**012** | .464 | 4.572 |
|  |  |  | With Stereopsis | .878 | 1.621 | .851 | -2.976 | 4.733 |
|  |  | With Stereopsis | Control | 1.640 | 1.562 | .547 | -2.074 | 5.354 |
|  |  |  | Without Stereopsis | -.878 | 1.621 | .851 | -4.733 | 2.976 |
| ***Total images*** | Tukey HSD | Control | Without Stereopsis | -1.990 | .622 | **.005** | -3.469 | -.510 |
|  |  |  | With Stereopsis | -.247 | 1.125 | .974 | -2.922 | 2.427 |
|  |  | Without Stereopsis | Control | 1.990 | .622 | **.005** | .510 | 3.469 |
|  |  |  | With Stereopsis | 1.742 | 1.167 | .299 | -1.034 | 4.518 |
|  |  | With Stereopsis | Control | .247 | 1.125 | .974 | -2.427 | 2.922 |
|  |  |  | Without Stereopsis | -1.742 | 1.167 | .299 | -4.518 | 1.034 |
| ***Horizontal Adjustment illusory images*** | Tukey HSD | Control | Without Stereopsis | -2.347 | .845 | **.018** | -4.356 | -.338 |
| Continue |  |  |  |  |  |  |  |  |
| ***Horizontal Adjustment illusory images*** | Tukey HSD | Control | With Stereopsis | -1.372 | 1.528 | .643 | -5.005 | 2.261 |
|  |  | Without Stereopsis | Control | 2.347 | .845 | **.018** | .338 | 4.356 |
|  |  |  | With Stereopsis | .975 | 1.586 | .812 | -2,795 | 4.746 |
|  |  | With Stereopsis | Control | 1.372 | 1.528 | .643 | -2.261 | 5.005 |
|  |  |  | Without Stereopsis | -.975 | 1.586 | .812 | -4.746 | 2.795 |
| ***Horizontal Adjustment images*** | Tukey HSD | Control | Without Stereopsis | -1.818 | .660 | **.019** | -3.389 | -.247 |
|  |  |  | With Stereopsis | -.233 | 1.194 | .979 | -3.074 | 2.607 |
|  |  | Without Stereopsis | Control | 1.818 | .660 | **.019** | .247 | 3.389 |
|  |  |  | With Stereopsis | 1.584 | 1.240 | .411 | -1.364 | 4.533 |
|  |  | With Stereopsis | Control | .233 | 1.194 | .979 | -2.607 | 3.074 |
|  |  |  | Without Stereopsis | -1.584 | 1.240 | .411 | -4.533 | 1.364 |
| ***Vertical Adjustment neutral images*** | Games-Howell | Control | Without Stereopsis | -1.633 | .972 | .223 | -3.984 | .716 |
|  |  |  | With Stereopsis | 1.383 | .706 | .163 | -.484 | 3.251 |
|  |  | Without Stereopsis | Control | 1.633 | .972 | .223 | -.716 | 3.984 |
|  |  |  | With Stereopsis | 3.017 | 1.083 | .**022** | .371 | 5.662 |
|  |  | With Stereopsis | Control | -1.383 | .706 | .163 | -3.251 | .484 |
|  |  |  | Without Stereopsis | -3.017 | 1.083 | **.022** | -5.662 | -.371 |
| ***Vertical Adjustment illusory images*** | Tukey HSD | Control | Without Stereopsis | -2.689 | 1.008 | **.024** | -5.088 | -.291 |
|  |  |  | With Stereopsis | -1.907 | 1.824 | .550 | -6.245 | 2.429 |
|  |  | Without Stereopsis | Control | 2.689 | 1.008 | **.024** | .291 | 5.088 |
|  |  |  | With Stereopsis | .781 | 1.893 | .910 | -3.719 | 5.283 |
|  |  | With Stereopsis | Control | 1.907 | 1.824 | .550 | -2.429 | 6.245 |
|  |  |  | Without Stereopsis | -.781 | 1.893 | .910 | -5.282 | 3.719 |

Continue

| ***Vertical Adjustment images*** | Tukey HSD | Control | Without Stereopsis | -2.161 | .707 | **.008** | -3.844 | -.479 |
| --- | --- | --- | --- | --- | --- | --- | --- | --- |
|  |  |  | With Stereopsis | -.262 | 1.279 | .977 | -3.304 | 2.780 |
|  |  | Without Stereopsis | Control | 2.161 | .707 | **.008** | .479 | 3.844 |
|  |  |  | With Stereopsis | 1.899 | 1.328 | .329 | -1.258 | 5.057 |
|  |  | With Stereopsis | Control | .262 | 1.279 | .977 | -2.780 | 3.304 |
|  |  |  | Without Stereopsis | -.1.899 | 1.328 | .329 | -5.057 | 1.258 |
