# Supplementary material for: Study of Geometric Illusory Visual Perception – A New Perspective in the Functional Evaluation of Children With Strabismus
Source: Front Hum Neurosci. 2022 Apr 13;16:769412. doi: 10.3389/fnhum.2022.769412 (PMC9043129; doi:10.3389/fnhum.2022.769412)
Supplement: Supplementary file 7 [file Table_7.DOCX]

**Table S7:** **Influence of the presence of amblyopia on the estimation of image size (mm) and response time (in seconds) between Groups: strabismic patients with amblyopia versus non-amblyopic individuals with strabismus**. Key: * Degrees of Freedom = 43; *Diff,* Difference in measuring image size (in milimeters); *Δ t,* Latency time to adjust the image (in seconds); M mean; SD, standart deviation; Med, Median.

|  |  |  | **Strabismic Patients (n 45)** | |  |  |  |
| --- | --- | --- | --- | --- | --- | --- | --- |
|  |  |  | **Amblyopia** | |  |  |  |
|  |  |  | **No**  **(n 37)** | **Yes**  **(n 8)** |  | **Teste t *** | |
| **Ajustament images presented** | **Test** |  | **M (SD)** | **M (SD)** | **Levene Test** | **t** | **p-value** |
| Neutral images | Vertical-Horizontal | *Diff* | 7.070 (25.542) | 5.604 (19.608) | .264 | .152 | .880 |
|  |  | *Δ t* | 8.362 (5.633) | 5.183 (2.949) | .086 | 1.541 | .131 |
|  | Brentano | *Diff* | 5.045 (26.274) | 11.897 (27.751) | .972 | -.663 | .511 |
|  |  | *Δ t* | 7.596 (2.792) | 6.946 (1.734) | .278 | -.205 | .839 |
|  | Ponzo | *Diff* | -4.423 (8,051) | 2.228 (9.578) | .888 | -2.051 | **.046** |
|  |  | *Δ t* | 6.251 (3.658) | 6.552 (2.832) | .422 | -.219 | .828 |
| Illusory images | Vertical-Horizontal | *Diff* | -151.559 (102.946) | -160.996 (121,993) | .951 | .228 | .821 |
|  |  | *Δ t* | 11.700 (4.247) | 9.452 (2.488) | .034 | 2.001 | .061 |
|  | Brentano | *Diff* | -1.643 (33.136) | -8.234 (40.269) | .627 | .491 | .626 |
|  |  | *Δ t* | 9.147 (3.145) | 8.042 (2.358) | .544 | -.050 | .960 |
|  | Ponzo | *Diff* | -72.634 (31.123) | -75.040 (23.508) | .250 | .206 | .838 |
|  |  | *Δ t* | 11.376 (5.459) | 11.149 (2.835) | .282 | .114 | .910 |
| Total images | Vertical-Horizontal | *Diff* | 98.682 (69.617) | -105.462 (76.330) | .813 | .246 | .807 |
|  |  | *Δ t* | 10.587 (4.175) | 8.029 (2.171) | .040 | 2.484 | **.022** |
|  | Brentano | *Diff* | .585 (28.147) | -1.524 (35.560) | .588 | .184 | .855 |
|  |  | *Δ t* | 8.630 (2.961) | 7.676 (2.054) | .465 | -.100 | .921 |
|  | Ponzo | *Diff* | -38.529 (17.153) | -36.405 (9.193) | .041 | -.493 | .627 |
|  |  | *Δ t* | 8.813 (3.735) | 8.851 (2.587) | .236 | -.027 | .979 |
| Horizontal Adjustment neutral images | Vertical-Horizontal | *Diff* | 7.418 (30.434) | .450 (23.514) | .544 | .608 | .547 |
|  |  | *Δ t* | 7.629 (5.046) | 5.233 (3.149) | .078 | 1.283 | .206 |
|  | Brentano | *Diff* | -3.855 (40.359) | -6.552 (31.385) | .474 | .177 | .860 |
|  |  | *Δ t* | 7.407 (3.100) | 6.392 (1.782) | .096 | .464 | .645 |
|  | Ponzo | *Diff* | -4.693 (13.381) | -1.715 (15.668) | .793 | -.554 | .582 |
|  |  | *Δ t* | 6.253 (3.837) | 6.706 (3.519) | .189 | -.307 | .761 |
| Horizontal Adjustment illusory images | Vertical-Horizontal | *Diff* | -98.783 (97.542) | -121.356 (152.167) | .256 | .534 | .596 |
|  |  | *Δ t* | 11.315 (4.057) | 9.617 (3.099) | .253 | 1.111 | .273 |
|  | Brentano | *Diff* | -11.098 (42.087) | -12.808 (75.178) | .366 | .089 | .929 |
|  |  | *Δ t* | 9.241 (3.248) | 8.361 (2.945) | .761 | -.274 | .786 |
|  | Ponzo | *Diff* | -81.512 (38.547) | -88.885 (28.218) | .180 | .510 | .613 |
|  |  | *Δ t* | 11.605 (5.097) | 10.789 (2.097) | .079 | .441 | .661 |
|  |  |  |  |  |  |  |  |
| Continue |  |  |  |  |  |  |  |
| Horizontal Adjustment images | Vertical-Horizontal | *Diff* | -63.382 (65.527) | -80.754 (100.320) | .238 | .616 | .541 |
|  |  | *Δ t* | 10.086 (3.709) | 8.156 (2.586) | .147 | 1.394 | .171 |
|  | Brentano | *Diff* | -8.684 (36.103) | -10.722 (59.716) | .531 | .128 | .899 |
|  |  | *Δ t* | 8.630 (3.066) | 7.704 (2.446) | .387 | -.046 | .964 |
|  | Ponzo | *Diff* | -43.103 (22.265) | -45.300 (11.380) | .033 | .404 | .690 |
|  |  | *Δ t* | 8.929 (3.982) | 8.748 (2.339) | .047 | .172 | .865 |
| Vertical Adjustment neutral images | Vertical-Horizontal | *Diff* | 6.721 (32.646) | 10.759 (28.904) | .748 | -.323 | .748 |
|  |  | *Δ t* | 9.095 (7.154) | 5.133 (3.229) | .063 | 1.522 | .135 |
|  | Brentano | *Diff* | 13.946 (32.323) | 30.346 (37.878) | .708 | -1.263 | .213 |
|  |  | *Δ t* | 7.785 (2.833) | 7.500 (2.359) | .521 | -.868 | .390 |
|  | Ponzo | *Diff* | -4.154 (9.287) | 6.173 (6.257) | .253 | -2.988 | **.005** |
|  |  | *Δ t* | 6.248 (5.528) | 6.399 (2.885) | .582 | -.075 | .941 |
| Vertical Adjustment illusory images | Vertical-Horizontal | *Diff* | -204.335 (120.085) | -200.635 (107.029) | .412 | -.080 | .936 |
|  |  | *Δ t* | 12.085 (4.906) | 9.287 (2.344) | .039 | 2.419 | .024 |
|  | Brentano | *Diff* | 7.810 (50.168) | -3.661 (35.545) | .204 | .612 | .544 |
|  |  | *Δ t* | 9.053 (3.186) | 7.723 (1.834) | .422 | .183 | .855 |
|  | Ponzo | *Diff* | -63.756 (31.263) | -61.195 (31.490) | .964 | -.210 | .835 |
|  |  | *Δ t* | 11.147 (6.316) | 11.509 (4.495) | .804 | -.153 | .879 |
| Vertical Adjustment images | Vertical-Horizontal | *Diff* | -133.983 (81.742) | -130.170 (66.197) | .233 | -.123 | .903 |
|  |  | *Δ t* | 11.088 (4.972) | 7.902 (1.985) | .042 | 2.957 | **.006** |
|  | Brentano | *Diff* | 9.856 (40.796) | 7.674 (31.914) | .404 | .142 | .888 |
|  |  | *Δ t* | 8.633 (2.945) | 7.648 (1.788) | .583 | -.151 | .880 |
|  | Ponzo | *Diff* | -33.955 (16.995) | -27.5109 (14.578) | .425 | -.994 | .326 |
|  |  | *Δ t* | 8.697 (4.308) | 8.954 (3.310) | .595 | -.158 | .875 |
