# Supplementary material for: Study of Geometric Illusory Visual Perception – A New Perspective in the Functional Evaluation of Children With Strabismus
Source: Front Hum Neurosci. 2022 Apr 13;16:769412. doi: 10.3389/fnhum.2022.769412 (PMC9043129; doi:10.3389/fnhum.2022.769412)
Supplement: Supplementary file 8 [file Table_8.DOCX]

**Table S8. Influence of the presence of vertical strabismus associated with horizontal strabismus to estimate image size (mm) and response time (in seconds) between Groups: Strabismus with associated vertical deviation versus Strabismus without associated vertical deviation.** Key: * Degrees of Freedom = 43; *Diff,* Difference in measuring image size (in milimeters); *Δ t,* Latency time to adjust the image (in seconds); M mean; SD, standart deviation; Med, Median.

|  |  |  | **Strabismic Patients (n 45)** | |  |  |  |
| --- | --- | --- | --- | --- | --- | --- | --- |
|  |  |  | **Vertical Deviation** | |  |  |  |
|  |  |  | **No**  **(n 19)** | **Yes**  **(n 26)** |  | **Test t *** | |
| **Ajustament images presented** | **Test** |  | **M (SD)** | **M (SD)** | **Levene**  **Test** | **t** | **p-value** |
| Neutral images | Vertical-Horizontal | *Diff* | 12.328 (18.764) | 2.776 (27.461) | .186 | 1.308 | .198 |
|  |  | *Δ t* | 6.507 (3.962) | 8.739 (6.107) | .074 | -1.391 | .171 |
|  | Brentano | *Diff* | -1.959 (27.039) | 12.279 (24.631) | .871 | -1.837 | .073 |
|  |  | *Δ t* | 7.374 (1.985) | 7.823 (3.037) | .258 | -.562 | .577 |
|  | Ponzo | *Diff* | -2.359 (7.501) | -3.885 (9.441) | .330 | .583 | .563 |
|  |  | *Δ t* | 6.115 (3.568) | 6.443 (3.511) | .600 | -.308 | .760 |
| Illusory images | Vertical-Horizontal | *Diff* | -160.911 (126.131) | -147.628 (88.991) | .157 | -.415 | .680 |
|  |  | *Δ t* | 10.882 (3.203) | 11.606 (4.628) | .063 | -.586 | .561 |
|  | Brentano | *Diff* | -12.641 (34.124) | 4.364 (32.881) | .946 | -1.687 | .099 |
|  |  | *Δ t* | 9.016 (2.632) | 9.261 (3.333) | .462 | -.265 | .792 |
|  | Ponzo | *Diff* | -64.630 (23.930) | -79.224 (32.331) | .060 | 1.661 | .104 |
|  |  | *Δ t* | 11.248 (4.986) | 11.400 (5.221) | .535 | -.099 | .922 |
| Total images | Vertical-Horizontal | *Diff* | -103.164 (83.645) | -97.493 (59.757) | .156 | -.266 | .792 |
|  |  | *Δ t* | 9.423 (2.760) | 10.650 (4.691) | .023 | -1.099 | .278 |
|  | Brentano | *Diff* | -9.080 (29.238) | 7.000 (27.694) | .903 | -1.879 | .067 |
|  |  | *Δ t* | 8,469 (2.318) | 8.782 (3.182) | .322 | -.363 | .718 |
|  | Ponzo | *Diff* | -33.495 (12.824) | -41.554 (17.367) | .052 | 1.709 | .095 |
|  |  | *Δ t* | 8.681 (2.724) | 8.912 (4.073) | .098 | -.223 | .825 |
| Horizontal Adjustment neutral images | Vertical-Horizontal | *Diff* | 6.936 (24,150) | 5.626 (32.873) | .115 | .147 | .884 |
|  |  | *Δ t* | 6.119 (3.782) | 7.995 (5.394) | .157 | -1.299 | .201 |
|  | Brentano | *Diff* | -6.871 (38.345) | -2.481 (39,448) | .992 | -.373 | .711 |
|  |  | *Δ t* | 6.704 (2.168) | 7.759 (3.257) | .166 | -1.225 | .228 |
|  | Ponzo | *Diff* | -3.753 (12.176) | -4.464 (14.897) | .335 | .170 | .866 |
|  |  | *Δ t* | 5.685 (3.186) | 6.808 (4.106) | .129 | -.992 | .327 |
| Horizontal Adjustment illusory images | Vertical-Horizontal | *Diff* | -111.832 (142.318) | -96.192 (75.017) | .014 | -.478 | .666 |
|  |  | *Δ t* | 10.670 (3.321) | 11.264 (4.364) | .115 | -.497 | .622 |
|  | Brentano | *Diff* | -11.179 (59.179) | -11.565 (40.154) | .221 | .026 | .979 |
|  |  | *Δ t* | 9.351 (2.907) | 9.265 (3.378) | .614 | .090 | .929 |
|  | Ponzo | *Diff* | -75.325 (30.475) | -88.303 (40.425) | .061 | 1.175 | .246 |
|  |  | *Δ t* | 11.404 (4.396) | 11.501 (4.993) | .475 | -.067 | .947 |
| Continue |  |  |  |  |  |  |  |
| Horizontal Adjustment images | Vertical-Horizontal | *Diff* | -72.242 (94.485) | -62.252 (51.076) | .016 | -.457 | .679 |
|  |  | *Δ t* | 9.153 (3.061) | 10.174 (3.932) | .239 | -.942 | .352 |
|  | Brentano | *Diff* | -9.743 (49.640) | -8.537 (33.192) | .209 | -.098 | .923 |
|  |  | *Δ t* | 8.469 (2.454) | 8.763 (3.224) | .326 | -.333 | .741 |
|  | Ponzo | *Diff* | -39.539 (17.648) | -46.383 (22.507) | .168 | 1.100 | .277 |
|  |  | *Δ t* | 8.545 (2.942) | 9.154 (4.239) | .145 | -.538 | .593 |
| Vertical Adjustment neutral images | Vertical-Horizontal | *Diff* | 17.721 (25.525) | -.074 (34.128) | .627 | 1.913 | .062 |
|  |  | *Δ t* | 6.895 (4.583) | 9.484 (7.917) | .026 | -1.380 | .175 |
|  | Brentano | *Diff* | 2.952 (34,709) | 27.027 (29.241) | .635 | -2.521 | **.016** |
|  |  | *Δ t* | 8.044 (2.430) | 7.887 (3.076) | .349 | .184 | .855 |
|  | Ponzo | *Diff* | -.964 (9.338) | -3.307 (9.901) | .591 | .803 | .427 |
|  |  | *Δ t* | 6.544 (6.790) | 6.078 (3.613) | .689 | .298 | .767 |
| Vertical Adjustment illusory images | Vertical-Horizontal | *Diff* | -209.990 (123.009) | -199.064 (114.147) | .473 | -.307 | .760 |
|  |  | *Δ t* | 11.094 (3.703) | 11.948 (5.297) | .056 | -.603 | .550 |
|  | Brentano | *Diff* | -14.103 (35,829) | 20.294 (50.683) | .060 | -2.529 | **.015** |
|  |  | *Δ t* | 8.681 (2.490) | 9.256 (3.417) | .377 | -.622 | .537 |
|  | Ponzo | *Diff* | -53.936 (27.197) | -70.144 (32.231) | .297 | 1.777 | .083 |
|  |  | *Δ t* | 11.091 (6.232) | 11.299 (5.928) | .854 | -.114 | .910 |
| Vertical Adjustment images | Vertical-Horizontal | *Diff* | -134.086 (82.658) | -132.734 (77.022) | .514 | -.056 | .955 |
|  |  | *Δ t* | 9.694 (2.764) | 11.127 (5.743) | .003 | -1.108 | .275 |
|  | Brentano | *Diff* | -8.418 (30,683) | 22.539 (39.804) | .166 | -2.828 | **.007** |
|  |  | *Δ t* | 8.469 (2.279) | 8.800 (3.226) | .283 | -.383 | .704 |
|  | Ponzo | *Diff* | -27.450 (14.482) | -36.725 (17.251) | .220 | 1.903 | .064 |
|  |  | *Δ t* | 8.818 (4.017) | 8.689 (4.265) | .564 | .103 | .919 |
